# Surfactant Protein D in Respiratory and Non-Respiratory Diseases

**DOI:** 10.3389/fmed.2018.00018

**Published:** 2018-02-08

**Authors:** Grith L. Sorensen

**Affiliations:** ^1^Institute of Molecular Medicine, University of Southern Denmark, Odense, Denmark

**Keywords:** surfactant protein D, respiratory distress syndrome, allergic asthma, chronic obstructive lung disease, atherosclerosis

## Abstract

Surfactant protein D (SP-D) is a multimeric collectin that is involved in innate immune defense and expressed in pulmonary, as well as non-pulmonary, epithelia. SP-D exerts antimicrobial effects and dampens inflammation through direct microbial interactions and modulation of host cell responses *via* a series of cellular receptors. However, low protein concentrations, genetic variation, biochemical modification, and proteolytic breakdown can induce decomposition of multimeric SP-D into low-molecular weight forms, which may induce pro-inflammatory SP-D signaling. Multimeric SP-D can decompose into trimeric SP-D, and this process, and total SP-D levels, are partly determined by variation within the SP-D gene, *SFTPD*. SP-D has been implicated in the development of respiratory diseases including respiratory distress syndrome, bronchopulmonary dysplasia, allergic asthma, and chronic obstructive pulmonary disease. Disease-induced breakdown or modifications of SP-D facilitate its systemic leakage from the lung, and circulatory SP-D is a promising biomarker for lung injury. Moreover, studies in preclinical animal models have demonstrated that local pulmonary treatment with recombinant SP-D is beneficial in these diseases. In recent years, SP-D has been shown to exert antimicrobial and anti-inflammatory effects in various non-pulmonary organs and to have effects on lipid metabolism and pro-inflammatory effects in vessel walls, which enhance the risk of atherosclerosis. A common *SFTPD* polymorphism is associated with atherosclerosis and diabetes, and SP-D has been associated with metabolic disorders because of its effects in the endothelium and adipocytes and its obesity-dampening properties. This review summarizes and discusses the reported genetic associations of SP-D with disease and the clinical utility of circulating SP-D for respiratory disease prognosis. Moreover, basic research on the mechanistic links between SP-D and respiratory, cardiovascular, and metabolic diseases is summarized. Perspectives on the development of SP-D therapy are addressed.

## Surfactant Protein D (SP-D)

Surfactant protein D is a pattern-recognition molecule belonging to the collectin family, a group of *col*lagen-containing C-type *lectins*. Human collectins also include surfactant protein A (SP-A), which has a tissue distribution and functions partially overlapping with those of SP-D. One main effect of SP-D is the aggregation and enhancement of phagocytosis of microbes and dying host cells. An additional classical member of the collectin family is the serum protein, mannan-binding lectin (MBL), which evokes complement activation through the lectin pathway in a complex with MBL-associated serine proteases (MASPs) (1). Moreover, three novel human defense collagens: collectin-10 (CL-10) [collectin liver 1, CL-L1, or CL-10], collectin-11 (CL-11) (collectin kidney 1, CL-K1, or CL-11), and collectin-12 (CL-12) (collectin placenta 1, CL-P1, or CL-12) have been identified (2–4). CL-10 and CL-11, partly in heterocomplexes with one another, are found in the circulation associated with MASPs and can induce complement activation (5). Different from the other collectins, CL-12 is a type II membrane protein with a fluid phase variant that has scavenger receptor, as well as complement activation, functions (6, 7).

Pulmonary surfactant is a multimolecular complex consisting of phospholipids and cholesterol (total 90%) and surfactant proteins (10%). The surfactant proteins consist of the high-molecular weight (HMW) hydrophilic proteins, SP-A and SP-D, and the low-molecular weight (LMW) and extraordinarily lipophilic surfactant protein B (SP-B) and surfactant protein C (SP-C), which are essential for the biophysical properties of surfactant phospholipids (8).

## Sites of SP-D Synthesis

Surfactant protein D has been localized to both the lung and non-pulmonary tissues. The protein is associated with external or luminal surfaces of the respiratory, digestive, glandular, reproductive tract, urinary, and vascular epithelia and glands (Table 1). This is consistent with the role of SP-D in pattern recognition, as the majority of locations in which it is expressed are at interfaces with the external milieu or with plasma, urine, tears, cerebrospinal fluid, and amniotic fluid, where the maintenance of a sterile milieu is critical.

**Table 1:** Detection of SP-D immunoreactivity or SP-D gene (*SFTPD*) expression in human organ systems.

Organ system	Localization	Technique
**Respiratory system**	Trachea	RT-PCR (9)
	Basal and intermediate tracheal epithelial cells	IHC (10)
	Tracheal glands	IHC (10)
	Lung	RT-PCR (9)
	Bronchial glands	IHC (10)
	Type II pneumocytes	IHC (9, 10)
	Club (Clara) cells	IHC (9)
	Alveoler airspace surface	IHC (10)
	Eustacian tube	WB of lavage (11)
	Seromucinous glands in sinonasal mucosa	IHC (12)
	Sinonasal mucosa	RT-PCR (13, 14)
		WB of lavage (13, 14)
		IHC (13)
	Bronchoalveolar lavage	ELISA (15); reviewed in Ref. (16)
		WB (17–19)
**Integumentary system**	Skin	RT-PCR (20, 21)
	Basal cells of epidermis	IHC (9, 10)
	Sebaceous glands	IHC (10)
	Eccrine sweat glands	IHC (9, 10)
	Hair shafts	IHC (21)
	Stratum spinosum in atopic dermatitis and psoriasis	IHC (20)
**Digestive system**	Von Ebner’s gland of the tongueTongue muscle	IHC (10)
	Parotid glandSubmandilar gland	RT-PCR (9, 22)
	Parotid gland and submandilar gland	WB of saliva from specific glands (22)
		IHC (9, 10, 22)
	Saliva	WB (23)
		ELISA (23)
	Eosophagal epithelium	IHC (9)
	Eosophagal striated muscle cells	IHC (10)
	Eosophageal glands	IHC (10)
	Stomach	RT-PCR (9)
	Parietal cells of the stomach	IHC (10)
	Body and pyloric gastric mucosa	IHC (9)
	Small intestine	RT-PCR (9)
	Crypts of Lieberkuhn	Immunohistoschemistry (10)
	Small intestinal mucosa	IHC (9)
	Liver	RT-PCR (9)
	Hepatocytes	IHC (10, 24)
	Gall bladder epithelium	IHC (9)
	Intra- and extrahepatic bile ducts	IHC (9, 10)
	Pancreas	RT-PCR (9)
	Intercalated ducts of pancreatic acini	IHC (9, 10)
**Urinary system**	Kidney	RT-PCR (9, 25)
		WB (11, 25)
	Renal tubular epithelium	IHC (10, 25)
	Podocytes of the glomeruli	IHC (10)
	Collecting ducts of kidney	IHC (9)
	Urether	IHC (9, 10)
	Urinary bladder epithelium	WB (26)
		IHC (9, 26)
**Reproductive system**	Oviduct epithelium	IHC (27)
	Uterus	RT-PCR (9)
	Secretory endometrium	IHC (27, 28)
	Cervical tissue	RT-PCR (27)
		WB (27)
	Cervical glands	*In situ* hybridization (27)
		IHC (10, 27, 28)
	Stratified squamous epithelium of the vagina	(28)
	Epithelium of the fallopian tube	(28)
	Theca interna cells of ovarian follicles	(28)
	Theca-lutein and granulosa cells of the corpus luteum	(28)
	Placenta	RT-PCR (9, 29)
		WB (29)
	Amniotic epithelium	IHC (30)
	Chorio-decidual layers	IHC (30)
	Decidual cells including decidual stromal cells	RT-PCR (31)
		IHC (31)
	Cytotrophoblasts, intermediate trophoblasts, and syncytiotrophoblasts	IHC (28, 31, 32)
	Amniotic fluid	SDS-PAGE and amino acid analysis (28, 33, 34)
		ELISA (30, 34, 35)
		WB (34, 36)
		Atomic force microscopy (37)
	Testes	RT-PCR (9, 38, 39)
		WB (39)
		IHC (10)
		ELISA (39)
	Spermatogonia	IHC (38, 39)
	Spermatocytes	IHC (38, 39)
	Cells of Sertoli	IHC (38, 39)
	Cells of Leydig	IHC (38, 39)
	Spermatozoal secretion	WB (39)
	Prostate	RT-PCR (9, 39, 40)
		WB (40)
	Epithelial cells of prostatic glands	*In situ* hybridizationIHC (40)
		IHC (10, 40)
	Seminal vesicle	IHC (10)
**Nervous system**	Brain	RT-PCR (9)
	Brainstem, cerebellum, choroid plexus, subventricular cortex, pia mater, cerebrospinal fluid, pineal gland	RT-PCR (41)
	Brainstem, cerebellum, choroid plexus, the circle of Willis, subventricular cortex, leptomeninx, and cerebrospinal fluid	WB (41)
	Follicular stellate cells of anterior pituitary gland	IHC (10)
	Ependymal cells in the ventricular region around the hippocampus, dentate gyrus small pyramid cells, choroid plexus, pinealocytes	IHC (41)
	Cerebrospinal fluid	ELISA (41, 42)
	Cornea	RT-PCR (43)
	Corneal epithelial cells	RT-PCR (44–46)
		WB (44, 45)
		IHC (43)
	Corneal epithelial cell secretion	WB (45)
	Conjunctiva	RT-PCR (43)
		WB (43)
	Lacrimal gland	RT-PCR (43)
		WB (43)
		IHC (10)
	Nasolacrimal duct	RT-PCR (43)
		WB (43)
	Tear fluid	Dot blot (43)
		WB (45)
		ELISA (45)
**Circulatory system**	Myocardium	RT-PCR (9)
		IHC (10)
	Vascular endothelium	RT-PCR (47, 48)
		WB (47, 48)
		IHC (28, 32, 41, 43, 47–50)
	Coronary artery smooth muscle	RT-PCR (47)
		WB (47)
		IHC (47)
	Plasma/serum	ELISA (15); reviewed in Ref. (16)
**Glands**^**a**^	Mammary glands	RT-PCR (9)
		IHC (10)
	Adrenal gland	RT-PCR (9)
	Adrenal cortex	IHC (10)
	Thyroid gland	IHC (10)
**Other**	Hassal’s corpuscle of thymus	IHC (10)
	Spleen	RT-PCR (9)
	Organ of corti	WB of lavage (11)
	Adipose tissue	RT-PCR (51)
	Adipocytes	RT-PCR (51)

*^a^SP-D expression in some glands is categorized together with relevant organ systems*.

*RT-PCR, reverse transcription polymerase chain reaction; IHC, immunohistochemistry; WB, western blotting; ELISA, enzyme-linked immunosorbent assay; SDS-PAGE, sodium dodecyl sulfate-polyacrylamide gel electrophoresis; SP-D, surfactant protein D*.

Some SP-D expressing non-pulmonary sites may produce surfactant-like materials and phospholipidic lubrication is present at numerous distinct sites. A major function of SP-D in the lung is as regulator of pulmonary surfactant lipid levels and, although not investigated, it has been speculated that SP-D may also participate in phospholipid homeostasis at extrapulmonary sites (52). Moreover, SP-D is expressed in the muscle cells and endothelium of the cardiovascular system, where it is suggested to function as an inhibitor of inflammatory signaling (47, 48). The expression patterns of SP-D have frequently been validated by different observers or by the use of diverse techniques (Table 1). SP-D immunostaining has also been observed in infiltrating white blood cells; for example, in the lung (9) and placenta (32). Moreover, SP-D immune-staining has been detected in the phagolysosome compartment or as granular staining of the cell membrane (9). Ultrastructural studies have demonstrated the presence of SP-D in the endocytic compartment of rat alveolar macrophages, but not in biosynthetic organelles (53), indicating that SP-D is not produced by these inflammatory cells, but rather is taken up by endocytosis.

## Regulation of SP-D Expression

The proximal promoter of SP-D mediates cell type-restricted, basal, and glucocorticoid-stimulated promoter activities as demonstrated *in vitro* (54). The SP-D promoter was originally identified containing multiple potential *cis*-regulatory elements including half-site glucocorticoid response elements, a canonical AP-1 consensus, several AP-1-like sequences, E-box sequences, several C/EBP and PEA3 motifs, putative interferon response elements, FoxA-bindings sites, and a GT-containing regulatory element and regulatory roles for AP-1 (junB, junD, c-Jun, and c-Fos), FoxA1/2 and GT-box binding proteins were identified by mutational studies (55–57). It was suggested that the permissive glucocorticoid regulation of SP-D expression is caused by increased promoter occupancy of C/EBPβ (58). Furthermore, retinoblastoma protein is demonstrated to stimulate *SFTPD* gene activation by forming a complex with C/EBPs bound to the C/EBPβ consensus site in the *SFTPD* promoter (59). Moreover, the calcineurin/NFAT pathway was demonstrated to be active *in vitro* resulting in assembly of NFATs, AP-1, and TFF-1 in a transcriptional complex in the proximal promoter of mouse *SFTPD* (60). Mitogen-activated protein kinase (MAPK)-mediated upregulation of SP-D expression has been reported in human corneal epithelial cells (61) and in human lung epithelial cells, where the expressional regulation was mediated *via* signaling through JNK, a MAPK (62). The expression of SP-D in corneal epithelium was further inhibited by pharmacological inhibitors of toll-like receptor (TLR)4 and myeloid differentiation primary response gene 88 (MyD88) signaling (44). Tumor necrosis factor-α (TNF-α) significantly augmented the level of SP-D expression in primary coronary endothelial cells. Moreover, the basal level SP-D was reduced by nitric oxide (NO) synthase inhibitor l-NAME, inhibitor of phosphoinositide 3-kinases (PI3Ks) Wortmannin and inhibitor of MEK1 activation and the MAP kinase cascade PD 98059. Inversely, SP-D expression could be increased by DETA NONOate (donor of NO) or insulin (activator of PI3K/Akt) (63).

Surfactant protein D expression is developmentally regulated and further regulated by epigenetic allele-specific expression outside the lung (64). Dexamethasone treatment during culture of fetal lung explants increased SP-D mRNA and protein (54), maternal steroid treatment increased fetal serum SP-D (65), and *in vitro* and *in vivo* studies have confirmed regulation of SP-D expression by glucocorticoids and shown a dramatic increase prior to birth (66–69). Fetal lung maturation occurs on exposure to glucocorticoids with a simultaneous increase in expression of SP-D by lung epithelial cells (70, 71). *In vivo* studies have further demonstrated an increase in SP-D mRNA after pharmacological inhibition of dipeptidyl peptidase activity (72) and both mRNA and protein after a brief 95% oxygen exposure in rats (73), and mRNA and protein was markedly increased following mouse exposure to the cytokines interleukin (IL)-4 (74, 75), IL-13 (76), and TNF-α (77), whereas insulin is reported to inhibit SP-D expression in lung epithelial cell line (78).

In addition, estrogen positively regulates expression of SP-D in the mouse uterus (79). Progesterone, along with estrogen synergizes SP-D expression, however, when administered alone results in negative regulation (80). SP-D transcript levels increased sevenfold in the prostate of castrated rats suggesting negative regulation by testosterone (81), while testosterone suppression downregulated transcript levels of SP-D in murine testis (38). Moreover, serum SP-D levels increase in Turner syndrome patients treated with growth hormone (82).

## Effects of SP-D

The primary reported effects of SP-D include binding of bacteria, viruses, fungi, and, recently, helminthic parasites, for clearance *via* opsonization for phagocyte recognition (83–90). A detailed review of the numerous interactions of SP-D with pathogenic microbes was provided by Nayak et al. (91). SP-D can also bind to other biological or abiotic particles and participate in their clearance from the airways and potential additional sites. Hence, SP-D is known to aggregate allergens and aid in their removal (92, 93), to enhance clearance of genomic DNA and apoptotic material (94), to aggregate and remove particulate material (95), and has the capacity to affect the mouse intestinal microbiota under certain experimental conditions (96, 97). The lectin activity of human SP-D favors its interactions with microbial ligands glycosylated with a variety of saccharides, including *N*-acetylmannosamine (ManNAc) > mannose > fucose (36, 98); however, SP-D is also recognized to bind a wide range of inhaled pathogens and can bind saccharides as well as lipids and nucleic acids, with broad specificity, to initiate phagocytosis. The diversity of its ligand interactions were recently reviewed by Jakel et al. (99).

In addition to opsonization for phagocytosis, the antimicrobial effects of SP-D include aggregation (100–102), which may enhance the efficiency of neutrophil extracellular traps (103), bacterial and fungal cell-membrane lysis (104–106), neutralization of infectivity (107–109), or dampening of innate signaling evoked by microbe-derived ligands (110).

Surfactant protein D enhanced phagocytosis and additional antimicrobial activity is beneficial to the host; however, in some rare cases, SP-D binding to pathogens can be a risk factor contributing to deterioration of (murine) disease and increased pathogen burden, for example, of hypocapsular *Cryptococcus neoformans* (111, 112) and *Pneumocystis carinii* (113). Initial studies of SP-D interactions with *Mycobacterium tuberculosis* demonstrated reduced bacterial uptake by macrophages, whereas *in vivo* studies suggest that SP-D is dispensable for immune control of infection (114–116).

## SP-D-Mediated Cellular Activation

A wealth of data from *in vitro* studies demonstrate that SP-D modulates immune cell, epithelial cell, fibrocyte, and smooth muscle cell functions (Table 2).

**Table 2:** SP-D-mediated cellular effects.

Cell type	Main reported SP-D-mediated effects	Reference
Macrophages	Opsonization of pathogens, allergens, DNA, apoptotic cells, and nanoparticles for phagocytosis; actin polymerization and chemotaxis; induction of MMP-1/3/12, IL-6/10/12, and IFN-γ expression; reduction of allergen induced and modulation of LPS-induced NO and IL-12 production and CD14/TLR signaling	(84, 87, 93, 95, 114, 115, 117–132)
Monocytes	Opsonization of pathogens for phagocytosis; chemotaxis; induction of IL-6/10, TNF-α, and IFN-γ expression; inhibition of viral entry	(128, 133–137)
Neutrophils	Opsonization of pathogens for phagocytosis; chemotaxis; modulation of virus-induced respiratory burst	(84, 85, 135, 138–141)
Eosinophils	Inhibition of eotaxin-triggered chemotaxis and eosinophilic cationic protein degranulation; increased apoptosis in activated cells; reduced TGF-β1 production	(142–144)
Lymphocytes	Inhibition of T cell proliferation and activation; repression of TLRs activation and TNF-α, IFN-γ, Th17, and IL-6 expression; delayed apoptosis after short-term incubation; induction of apoptosis in activated lymphocytes after extended incubation	(137, 145–152)
Basophils	Inhibition of IgE binding to the allergens of *Aspergillus fumigatus* and hence blocking allergen-induced histamine release from basophils	(153)
Mast cells	Decreased allergen-induced IgE-dependent degranulation	(154)
Dendritic cells	Modulation of antigen presentation; induced uptake of particles or antigens; maintenance of DC-SIGN expression; reduced TNF-α secretion	(93, 95, 117, 150, 155–157)
NK-cells	Suggested stimulation of IFN-γ secretion	(134)
Epithelial cells	Suggested repression of fungal spore binding; repression of bacterial binding; increased allergen binding and uptake; neutralization of viral infectivity; inhibition of proliferation and migration of human lung adenocarcinoma cell line trough suppression of EGF signaling; inhibition of the expression of inflammatory cytokines through TLR4 signaling in corneal epithelial cells	(25, 26, 44, 46, 158–162)
Fibrocytes	Reduced TGF-β1 and CXCR4 expression	(163)
Smooth muscle cells	Repression of TNF-α- and LPS-induced IL-8 release	(47)

*NK-cells, natural killer cells; MMP-1/3/12, matrix metalloproteinase 1/3/12; IL-6/8/10/12, interleukin 6/8/10/12; IFN-γ, interferon-γ; LPS, lipopolysaccharide; NO, nitrogen oxide; CD14, cluster of differentiation 14; TLR (4), toll-like receptor (4); TNF-α, tumor necrosis factor-α; TGF-β1, transforming growth factor-β1; Th17, T helper 17 cell; IgE, Immunoglobulin E; DC-SIGN, dendritic cell-specific intercellular adhesion molecule-3-grabbing non-integrin; EGF, epidermal growth factor; CXCR4, C-X-C chemokine receptor type 4; SP-D, surfactant protein D*.

## SP-D Receptors

The identification of SP-D receptors is important for understanding its immune-regulatory and homeostatic functions in different cell types. A series of SP-D receptors, or receptor candidates, have been identified; however, the cellular effects of SP-D *via* some of these receptors have yet to be determined mechanistically and validated by independent research.

### Cluster of Differentiation 14 (CD14)/TLR/Myeloid Differentiation Factor 2 (MD-2)

Initially, SP-D was demonstrated to interact in a Ca^2+^-dependent manner with glycosylated CD14 *via* its C-type lectin domain (CTLD), thereby inhibiting lipopolysaccharide (LPS) binding (164). Subsequently, allergen-induced activation of macrophages and dendritic cells by SP-D mediated by suppression of the CD14/TLR signaling pathway was discovered (117). Furthermore, SP-D can inhibit the cell surface binding of LPS to TLR4/MD-2-expressing cells and attenuate MD-2 binding to LPS through the CTLD (118, 165, 166). Moreover, SP-D modulation of epithelial responses to additional microbial stimuli was recently documented as dependent on TLR4 and MyD88 (44). Such observations may partially explain the results of *in vivo* studies indicating that SP-D inhibits inflammation caused by bacterial LPS (167–169). SP-D can also bind to TLR2 (165).

### Signal-Regulatory Protein-α (SIRP-α)/Calreticulin/CD91

In a highly cited study from 2003, it was suggested that free SP-D binds to the cellular receptor, SIRP-α, through its CTLD, resulting in an inhibitory signal preventing activation of mononuclear phagocytes, nuclear factor-κB activation, and secretion of inflammatory cytokines through phospho-p38-dependent signaling. By contrast, when the CTLD is occupied by a ligand, SP-D was suggested to interact with a cell surface receptor complex consisting of CD91 and calreticulin, through its collagen-like domain, promoting inflammatory cell activation (170). However, the main focus of the study was SP-A interactions. Consequently, the evidence for direct SP-D/SIRP-α binding was weak and CD91/calreticulin interactions were only demonstrated for SP-A. The study inspired further research, which validated the binding of SP-D to N-glycosylated sites in the membrane-proximal domain of SIRPα, and to SIRPβ, a related SIRP (171). Moreover, in the absence of inflammation, SP-D can suppress the phagocytic function of alveolar macrophages by binding to SIRPα, thereby altering the activity of its downstream signaling effectors. By contrast, during LPS-induced inflammation, recruited mononuclear phagocytes partly escape SP-D-mediated inhibition and contribute to cell clearance (119) while IL-12p40 production is suppressed (120). Glucocorticoid treatment further relieves SP-D-driven suppression of apoptotic cell uptake through downregulation of SIRPα (172).

At the same time that SP-D interaction with SIRPα was validated, it was demonstrated that an interaction of SP-D with macrophage calreticulin appeared to be dependent on biochemical modification of SP-D. *S*-nitrosothiol (SNO)-SP-D, formed by nitrosylation of N-terminal cysteines in SP-D, but not native SP-D, was chemoattractive for macrophages, inducing downstream p38 phosphorylation. The authors suggested that SP-D acts to integrate the status of the lung lining, initiating inflammatory responses under various pathological conditions, through calreticulin-mediated signaling, while maintaining a quiescent state through SIRPα signaling in the absence of stress (173).

### Leukocyte-Associated Immunoglobulin-Like Receptor 1 (LAIR-1)

Leukocyte-associated immunoglobulin-like receptor 1, which is a receptor expressed on most immune cells, and for which collagens are high-affinity ligands, is an inhibitory SP-D receptor. The collagen stalk of SP-D is essential for the interaction with LAIR-1, which results in functional reduction of reactive oxygen species (ROS) signaling in a neutrophilic cell line (174). The authors therefore suggested that a lack of SP-D/LAIR-1 interaction could be responsible for the increased production of hydrogen peroxide in lung homogenates previously observed in SP-D deficient (*Sftpd^−/−^*) mice (175).

### Osteoclast-Associated Receptor (OSCAR)

Another collagen receptor, OSCAR, expressed in inflammatory C-C chemokine receptor type 2 (CCR2) + monocytes and macrophages, can functionally interact with multimeric (cruciform) SP-D, resulting in a pro-inflammatory response. This interaction leads to TNF-α release from CCR2^+^ monocytes and apparent internalization of the SP-D/OSCAR complex in alveolar macrophages (133). OSCAR is also expressed in osteoclasts, dendritic cells, and endothelial cells (176).

### Fc Receptor γII (FcγRII/CD32)

Surfactant protein D binding to FcγRII (CD32) on eosinophils has been detected by flow cytometric analysis and may explain the inhibitory effect of SP-D on IgG and serum-triggered eosinophilic cationic protein degranulation by eosinophils (142).

### NKp46

*Sftpd^−/−^* mice have reduced expression of pulmonary interferon-γ (IFN-γ) when exposed to ozone; therefore, it was hypothesized that IFN-γ-producing natural killer (NK) cells interact with SP-D through the glycosylated membrane receptor, NKp46. Indirect evidence for such an interaction came from the reduced binding between SP-D and NK cells obtained from *NKp46^−/−^* mice, relative to those from *NKp46^+/+^* mice; the authors of this study suggested that this interaction may be involved in the IFN-γ-dependent impaired dendritic cell homing to lymphoid tissue in *Sftpd^−/−^* mice (134).

### G Protein-Coupled Receptor 116 (GPR116)

The phenotype of GPR116 (Ig-Hepta) deficient mice is very similar to that of *Sftpd^−/−^* mice, including accumulation of surfactant lipids, enlarged alveoli, hypertrophy of type II alveolar (AT-II) cells, decreased surfactant uptake by type II alveolar cells (AT-II cells), accumulation of enlarged foamy macrophages, and enhanced expression of the matrix metalloproteinase 12 (*Mmp12*) gene. Therefore, it was hypothesized that this adhesion class of G protein-coupled receptor may interact with SP-D. GPR116 is highly expressed in type II pneumocytes and immunoprecipitation of flag-tagged recombinant proteins supports SP-D as a likely ligand of this receptor (177).

### Uroplakin Ia (UPIa)

Uroplakin Ia is a glycoprotein expressed on bladder urothelium that serves as a receptor for FimH, a lectin in bacterial pili, and this interaction initiates uropathogenic *Escherichia coli* (UPEC) infection. SP-D binds directly to UPIa, which is rich in high mannose glycans, and thereby inhibits the adherence and cytotoxicity of UPEC in a human bladder epithelial cell line. These *in vitro* observations were supported by the results of experiments demonstrating that exogenous administration of SP-D inhibited UPEC adherence to the bladder and dampened UPEC-induced inflammation in mice (26).

### Epidermal Growth Factor Receptor (EGFR)

Surfactant protein D binds directly to high mannose-type N-glycans in EGFR and the interaction blocks the binding of epidermal growth factor (EGF) to EGFR, suppressing EGF signaling and inhibiting the proliferation and migration of two human lung adenocarcinoma epithelial cell lines, indicating that lung cancer cells are regulated by SP-D *via* autocrine mechanisms (158, 178).

Additional receptor candidates, Jäkel and Sim, demonstrated that SP-D can bind to a 20- to 22-kDa structure on macrophages and dendritic cells in a calcium-dependent manner; however, they were unable to identify the nature of the structure (179). Interaction of SP-D with dendritic cell-specific intercellular adhesion molecule-3-grabbing non-integrin reduces SP-D-mediated HIV-1 capture and transfer to CD4+ T cells (180).

## Additional Interacting Host Molecules

Secreted host molecules, reported to bind SP-D include decorin (181); the protease inhibitor, alpha(2)-macroglobulin, which enhances bacterial agglutination and protects SP-D against elastase-mediated degradation (182); deleted in malignant brain tumor 1/gp340, which enhances SP-D-mediated viral aggregation (183, 184); and defensins, which, according to subtype, may cause SP-D to precipitate out of bronchoalveolar lavage (BAL) fluid or have additive viral neutralizing activity when combined with SP-D (185, 186). Moreover, various classes of immunoglobulin, including IgG, IgM, IgE, and secretory IgA, bind SP-D. SP-D aggregates immunoglobulin-coated beads and enhances their phagocytosis and IgM–SP-D complexes effectively opsonize late apoptotic cells and enhance their clearance by alveolar macrophages in the lungs (121, 187).

## SP-D Structure, Decomposition, and Proteolytic Degradation

**Figure 1:**
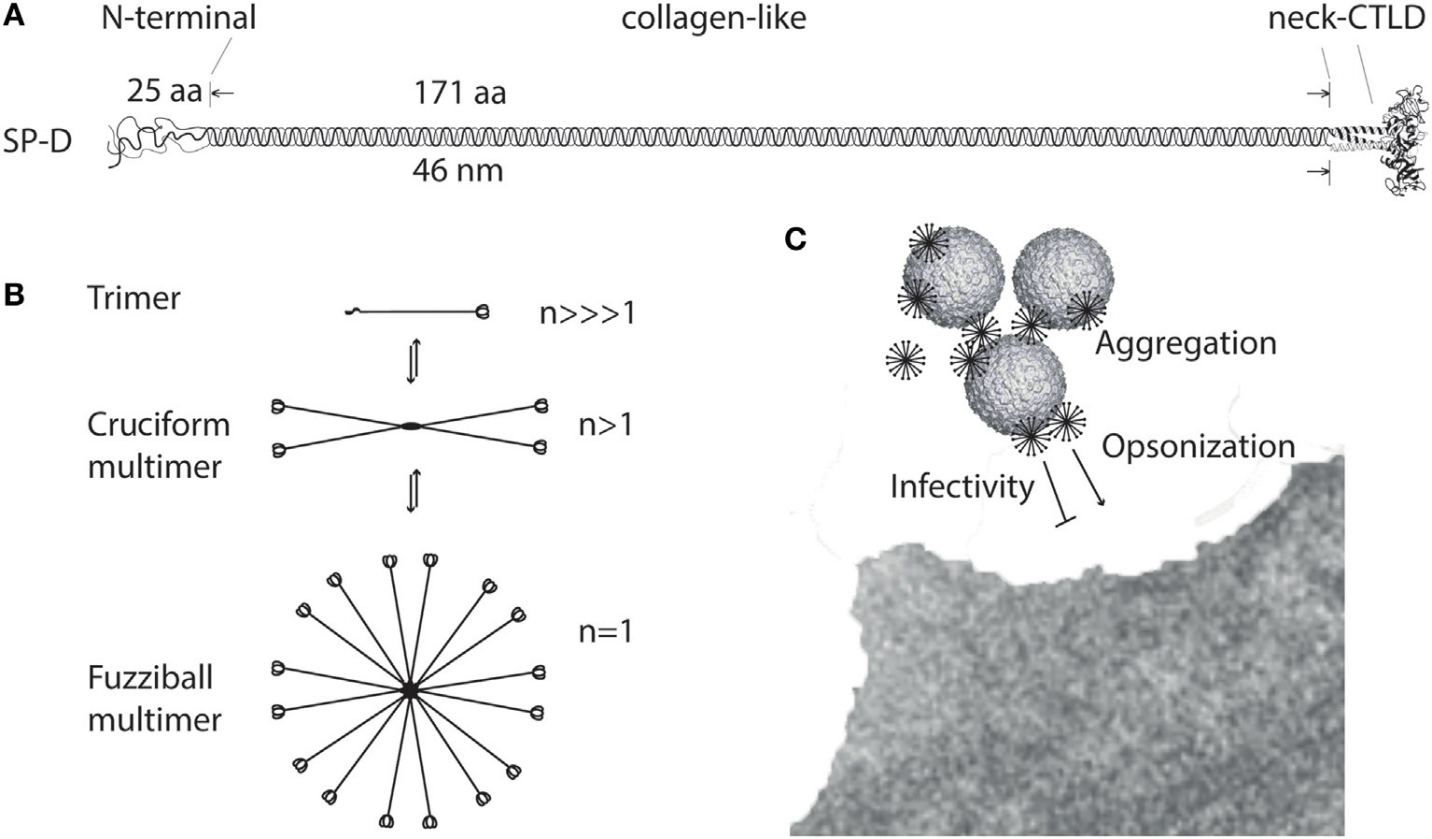
Multimerization of surfactant protein D (SP-D). **(A)** Regions of the trimeric SP-D subunit. The subunit structure has been drawn to the approximate dimensions of the protein domains. Adapted with permission from Ref. (190). **(B)** Multimerization of the trimeric SP-D subunit (3 chains) into 4-subunit cruciform (12 chains) or fuzziball >4-subunit (>12 chains) structures of SP-D. **(C)** Schematic overview of how multimeric SP-D is implicated in antimicrobial defense. Binding of multimeric SP-D to microbe-associated glycans may block interaction of the microbe with its receptors, aggregate the microbes, or SP-D may act as an opsonin, enhancing endocytic uptake of the microbe in host cells. Only fuzziball SP-D multimers are shown for simplicity. CTLD, C-type lectin domain.

The amino acid sequence (375 aa) of the mature SP-D monomer consists of four structural domains: (1) an N-terminal domain involved in intermolecular disulfide bond formation; (2) a collagen domain, important for spacing of the CTLDs; (3) an α-helical neck region involved in protein trimerization and spacing of the CTLDs; and (4) a globular C-terminal CTLD (Figure 1A), responsible for Ca^2+^-dependent binding of microbial ligands (188). The SP-D protein structure is stabilized by assembly into trimers and multimers *via* two conserved cysteine residues in the N-terminal domain (33, 189).

The individual “arm” length of an SP-D monomer is approximately 46 nm (188), making it a molecule with dimensions the same order of magnitude as certain viruses, as shown by electron microscopy (191). The molecular mass of SP-D molecules ranges from <100 to >1,000 kDa, as a consequence of different degrees of multimerization. Disulfide-bridge dependent trimerization of SP-D into subunits and further multimerization into cruciform or fuzziball structures (astral bodies), which can contain 32 (or more) trimeric subunits (17, 192) (Figure 1B), provides the spatial arrangement required for high-avidity interaction of the CTLDs with multivalent microbial ligands (85, 100). The effects of SP-D thus depend on the degree of multimerization for the binding of ligands (108, 115) (Figure 1C) or the cellular receptors evoking major SP-D antimicrobial effects.

## HMW and LMW SP-D

High-molecular weight SP-D multimers are only partly dependent on disulfide crosslinking of the N-termini, and a proportion of SP-D subunits are non-covalently associated. This allows interconversion between HMW SP-D and LMW SP-D trimers, as demonstrated using size permeation chromatography (36) (Figure 1B). The HMW/LMW ratio depends on the concentration of the protein in solution, with low-protein concentrations favoring the decomposition of multimers into trimers. In addition, the HMW/LMW ratio increases with affinity purification of SP-D, suggesting that ligand-binding facilitates assembly of SP-D trimers into multimers (36).

A single-nucleotide polymorphism (SNP), rs721917, in the SP-D gene (*SFTPD*) results in expression of either methionine or threonine at position 11 (Met11Thr) in the mature protein. The HMW/LMW ratio in body fluids appears to vary according to the amino acid at this position, with 1:(1–1.6) for methionine 11 (Met11) and 1:(3–5) for threonine 11 (Thr11) allelic variants (36, 37), as assessed by monoclonal immunodetection of SP-D CTLDs after size permeation chromatography. HMW SP-D exhibits markedly increased binding to a majority of microbial ligands and microbes (36, 37), which calls into question the role of LMW SP-D, other than as a large reservoir of subunits available for assembly of HMW SP-D when enriched on microbial surfaces; however, whereas HMW SP-D binds preferentially to intact influenza A virus and bacteria, trimeric SP-D favors Ca^2+^-independent binding to isolated bacterial LPS (37). Use of ManNAc-affinity chromatography, in place of traditional maltose-affinity chromatography, allowed preparation of enriched natural trimeric human SP-D, facilitating investigation of this molecule. Natural LMW SP-D also binds to endogenous circulating ligands including low-density lipoprotein (LDL), oxidized LDL (oxLDL), and high-density lipoprotein (HDL), in a partially Ca^2+^-independent manner, whereas HMW SP-D does not bind lipoproteins (36).

## *In Vivo* Studies of SP-D Size Variants

Transgenic *Sftpd^−/−^* mice expressing either the human SP-D Met11 or Thr11 allelic variants were generated to test the hypothesis that this allelic variation is implicated in disease; however, the expression of the allelic variants was under the control of a promoter for ubiquitous expression. Consequently, the distribution of SP-D levels in the lung and serum appeared to differ from that of endogenous SP-D. This made comparisons of effects with normal mice difficult, despite sustained allele-dependent HMW/LMW SP-D distribution. The low transgene expression levels in the lung generated pulmonary phenotypes in both transgenic mice somewhat resembling *Sftpd* deficiency, which is characterized by mild emphysema and the presence of foam cell-like macrophages (193–197). Alternative studies made point mutations affecting the N-terminal cysteines involved in the stabilization of SP-D HMW multimers, or deletions of the collagen region and/or the N-terminal region. Such studies demonstrated that trimeric SP-D subunits have the same saccharide selectivity as multimers, but appear to have a weaker and more restricted range of antimicrobial activity (85, 100, 198, 199). *In vivo* studies, where the N-terminal cysteine mutated SP-D was overexpressed, or an SP-D collagen deletion mutant protein expressed, in *Sftpd^−/−^* or *wild-type* mice demonstrated that native SP-D is essential for pulmonary phospholipid homeostasis and prevention of airspace enlargement (189, 200). Collectively, those studies suggested that multimerization is important; however, *in vivo* administration of repeated high doses of a 60-kDa recombinant trimeric fragment of SP-D [60-kDa recombinant trimeric fragment of SP-D lacking the N-terminal but retaining a part of the collagen region (rfhSP-D)], lacking the N-terminus but retaining part of the collagen region, appeared to have similar effects to native SP-D in reducing lipidosis, apoptotic macrophages, alveolar type II cell numbers, and airspace enlargement in mice (201, 202), suggesting that the homeostatic effects of SP-D are predominantly mediated by the CTLD, and that high therapeutic levels of trimeric human SP-D may compensate for a relatively low target avidity. Trimeric rfhSP-D has subsequently been used successfully in antimicrobial or anti-inflammatory therapy in a series of *in vivo* studies (110, 203–206) and can also induce apoptosis of activated immune cells (143, 145). However, rfhSP-D has also failed to demonstrate effects in some contexts (92) and the extent to which the effects of full-length multimeric SP-D can be mimicked by high levels of trimeric SP-D is not yet entirely clear.

## Genetic Variation Affecting SP-D Structure

According to a study of adult twins, genetic factors explain an estimated 83% of variation in constitutive serum SP-D levels. Moreover, the rs721917 *SFTPD* SNP is associated with serum levels of SP-D (207) and explains 39% of phenotypic variation (36), with the Met11 allelic variant associated with the highest levels (37). Both allelic variants were detected at relatively high frequencies (Thr11/Thr11 = 0.18, Met11/Thr11 = 0.43, Met11/Met11 = 0.36) in a North European population (208). Moreover, the rs721917 allelle frequency distribution is highly similar in different populations (209, 210), although ethnic differences are documented (211).

Consistent with genetic determination of SP-D levels, the constitutive distribution of HMW and LMW SP-D in human body fluids is also genetically determined, with individuals homozygous for the Met11 allele having a relative predominance of HMW SP-D and Thr11 allele homozygotes more LMW SP-D. The dependency of SP-D molecular size on rs721917 genetic variation is supported by the size distribution of recombinant SP-D expressed from the two allelic variants in a human cell line (37). The varying abilities of the allelic variants to assemble into multimers is postulated to be attributable to the different hydrophobic properties of Met and Thr, or partial O-linked glycosylation of the Thr11-residue, which may affect close-proximity disulfide bonding, thus limiting the stability of Thr11-variant multimers (34, 37).

Several studies have linked rs721917 with clinical pathologies (Table 3), which may indicate involvement of SP-D size variation in pathogenesis. Some rs721917 association studies have demonstrated a strong interaction with smoking, including in preclinical cardiovascular disease (CVD) (212–214). Moreover, some data are contradictory; for example, SNP-analysis associated rs721917 variation with chronic obstructive pulmonary disease (COPD) (215), whereas genome-wide association (GWA) analysis did not confirm this association, although several other *SFTPD* SNPs were identified as associated with COPD (216). Recently, an association of rs721917 variation with SP-D size variation was reported in respiratory disease (217); however, the overall conclusion from studies of rs721917 variant associations is that both allelic variants may be deleterious in different disease contexts, although the majority of studies suggest disease associations with the Thr11 allele.

**Table 3:** *SFTPD* rs721917 (Met11Thr) variation in disease.

Study	Population	Allele	Cases/controls	Reference
Acute RDS	German	No association	52/46	(218)
Allergic rhinitis	Chinese	Thr11	216/84	(219)
Asthma	German	No association	322/270	(220)
Atopy in asthma	Black	Met11	162/97	(221)
*Cardiovascular disease*				
Atherosclerosis, preclinical	Danish	Thr11	396 preclinical cases	(214)
Atherosclerosis, preclinical	Danish	Met11-smoking interaction	396 preclinical cases	(214)
Coronary stenosis	Norwegian	Thr11	130/100	(222)
Chronic lung allograft dysfunction with reduced survival	Columbian	Thr11	191 cases/NA	(223)
COPD	Mixed	Thr11^b^	389/472	(224)
COPD	Japanese	Thr11	188/82	(225)
COPD	Pakistani	Met11	115/106	(215)
COPD, survival, change in FEV_1_, positive bronchodilator response	Chinese	Thr11^a^	192/128	(226)
Lowest lung function in smokers	Danish	Thr11	492 smokers/1017 non-smokers	(212)
Emphysema	Japanese	Thr11	160/971	(225)
Community-acquired pneumonia				
Multi-organ dysfunction syndrome	Spanish	Thr11	178/1,186	(227)
Acute RDS	Spanish	Thr11	29/510	(227)
Cystic fibrosis, renal involvement	Spanish	Thr11	210/NA	(228)
Diabetes, type II	Spanish	Met11	440/2,270	(229)
Inflammatory bowel disease	American	No association	256/376	(230)
Interstitial pneumonia	Japanese	Thr11	93/1,249	(231)
Lung cancer	Japanese	Thr11	140/1,202	(231)
Prematurity				
Bronchopulmonary dysplasia	Greek	Thr11^a^	71 neonates	(232)
Diverse respiratory outcomes	Danish	Met11	202/211	(233)
RDS	German	No association	283 preterm infants	(234)
Preterm birth, spontanous	Finnish	Met11	406/201	(235)
Rheumatoid arthritis, erosive disease	Danish	Thr11	456/533	(213)
Respiratory syncytial virus infection	Mixed	Thr11^a^	148/NA	(236)
Respiratory syncytial virus infection	Finnish	Met11	84/93	(209)
Tuberculosis	Mexican	Thr11	178/101	(237)

*SFTPD Met11Thr allelic variant with association to increased risk are indicated*.

*COPD, chronic obstructive pulmonary disease; FEV_1_, forced expiratory volume in 1 s; Met11, methionine 11; RDS, respiratory distress syndrome; Thr11, threonine 11*.

*^a^Information derived from haplotype analysis*.

*^b^Genetic associations were attempted replicated in additional cohorts in the same study, but SFTPD Met11Thr association was only reported significant in 1 out of 4 cohorts*.

## Biochemical Modification of SP-D

Posttranslational modifications of SP-D include partial hydroxylation of proline and lysine residues in the collagen-like region. Furthermore, SP-D can undergo glycosyl-galactosyl O-linked glycosylation of hydroxylated lysine residues and O-linked glycosylation of N-terminal threonines. N-glycosylation of SP-D also occurs within the collagen-like region. These modifications are partial, hence the molecular weight of resulting molecules varies from 37 to 50 kDa, as assessed by sodium dodecyl sulfate-polyacrylamide gel electrophoresis (SDS-PAGE) and mass spectrometry (34, 188, 238–241). Some posttranslational SP-D modifications may alter its propensity for multimerization and a fraction of LMW SP-D purified from normal body fluid (late amniotic fluid) is unable to assemble into multimers (36). This fraction probably contains modified or partly degraded SP-D and appears to be enriched under inflammatory conditions.

### Glycosylation Variants

Surfactant protein D can undergo O-linked glycosylation of Thr11 and this variant is essentially present in the trimeric form (34). Moreover, endothelial SP-D appear with lower molecular weight band pattern in SDS-PAGE and may represent immature intracellular protein, or a posttranslationally modified version of SP-D, different from the forms produced by lung cells or cells that generate SP-D in amniotic fluid (214); however, this remains to be explored.

One type of disease-induced modification of SP-D is suggested to be increased levels of core fucose in the SP-D N-glycan. The N-glycan is not expected to affect the quaternary structure of SP-D and normal SP-D N-glycan comprises core fucose; however, the glycosylation is only partial (240) and depends on fucosyltransferase activity. Glycomic analysis demonstrated that the levels of core-fucosylated N-glycan in SP-D are increased, relative to total SP-D, in smoking COPD subjects, but not in non-smokers (241).

### Nitrosylation

The two N-terminal cysteines at positions 15 and 20 of the SP-D N-terminal region are implicated in inducible nitric oxide synthase (iNOS)- and NO-mediated control of multimerization of SP-D through formation of *S*-nitrosothiol SP-D (SNO-SP-D) (173). SNO-SP-D levels increase during inflammation, and its formation results in decomposition of SP-D multimers (173, 242–245). Radiation-induced lung injury is thus reported to result in iNOS-dependent disruption of SP-D multimers in mouse BAL (246). As described earlier, SNO-SP-D, but not SP-D (neither multimeric nor trimeric), is chemoattractive for macrophages and can induce cellular p38 phosphorylation, indicating that SNO-SP-D reacts differently with cellular receptors compared with SP-D (173).

### Oxidative Damage

Non-reducible crosslinking of HMW SP-D occurs in normal late amniotic fluid (36), in BAL from alveolar proteinosis (17), and in asthma patient BAL after provocation with an allergen (242). Formation of tyrosine-dependent covalent crosslinking within the neck/CTLD of SP-D is a result of reactive oxidant species (ROS), including peroxynitrite activity, and reduces SP-D ligand aggregation (247).

Additional oxidative damage is caused by neutrophil myeloperoxidase and its specific reactive oxidant product, hypochlorous acid. Hypochlorous acid can cause abnormal but reducible N-terminal disulfide crosslinking of SP-D, and although the mechanism is not fully elucidated, these modifications result in loss of the aggregating activity of SP-D *in vitro* and in the context of acute inflammation *in vivo* (248).

### Proteolytic Degradation

Surfactant protein D is subjected to diverse types of proteolytic degradation, resulting in the release of LMW breakdown products (<30 kDa). Various studies have demonstrated that SP-D can be fragmented in the human lung (249–252), and relevant enzymes include host proteases such as neutrophil elastase, cathepsin G, protease 3, and MMP-9, along with elastase produced by *Pseudomonas aeruginosa*, and house dust mite protease (253–261).

Clinically relevant proteolytic degradation of SP-D is observed in different settings, including acute lung injury (ALI) (262) and cystic fibrosis (CF) BAL. SP-D degradation by CF relevant proteases is well described; neutrophil elastase appears to be the most important contributor and reduces the CTLD lectin activity of SP-D *in vitro* (249, 250, 254, 263); however, the functional consequences of this have been questioned, as physiological concentrations of calcium can delay or abolish SP-D proteolysis (250, 254). Some studies have shown that, although present, the proteolytic fragmentation of SP-D does not appear to be responsible for reduced SP-D lectin activity in CF, which is instead suggested to be mediated by oxidative modifications (18). Nevertheless, SP-D levels are decreased in elastase-positive CF BAL samples, and the depressed levels are generally suggested to result from proteolytic activity (249, 250, 264). Clinical SP-D deficiency is not documented; however, BAL levels of SP-D may decrease during disease to levels that prevent immunodetection (264–266).

Surfactant protein D breakdown products are also detected in samples obtained from patients with severe asthma. In one recent study, no breakdown products were detected by western blotting (WB) in BAL, whereas the authors succeeded in identifying breakdown products in serum after a StrataClean Resin™ incubation step. The detection of breakdown products correlated with increased enzyme-linked immunosorbent assay (ELISA)-based detection of serum SP-D, at the expense of BAL SP-D. Hence, the ELISA readout may represent a mixture of complete and degraded SP-D forms (19). LMW SP-D breakdown products, together with trimeric SP-D, and non-reducible SP-D, can be separated by size permeation chromatography and WB, as demonstrated using BAL obtained from children with gastroesophageal reflux (267) and serum from patients with asthma (217).

## Determinants of SP-D Levels

Surfactant protein D is a hydrophilic molecule and a variety studies using disease selected cohorts have demonstrated that variation in levels of BAL or circulatory SP-D may be associated with pulmonary disease, as previously reviewed (16). Moreover, sex, smoking, adiposity, and age were reported as important determinants of constitutional levels of circulating SP-D in a homogenous Caucasian population (208); however, studies of age-induced changes in SP-D levels are contradictory. Rat studies have demonstrated an association between reduced alveolar SP-D levels with increased oxidative damage (268). By contrast, studies of human alveolar SP-D levels demonstrated no detectable change in its levels with aging (269), neither was induction of human nor mouse alveolar SP-D observed during aging, alongside induction of cytokines and oxidants (270). Although a clear age-related induction of circulating SP-D was reported in Danes (208), this relationship has not been identified in all ethnicities (271), and may not occur in the presence of disease (272, 273).

### Translocation of SP-D from Lung to Blood

**Figure 2:**
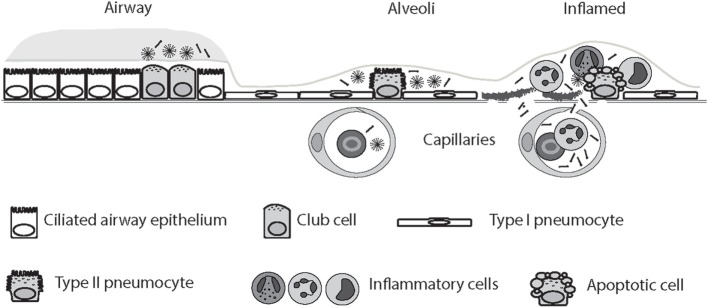
Circulatory spill-over of pulmonary surfactant protein D (SP-D) in inflammatory disease. SP-D is synthesized by Club cells, type II alveolar cells, and endothelial cells, and the levels of SP-D multimers and trimers in the serum are highly genetically determined. In the inflamed lung, the production of trimeric SP-D is increased, due to various chemical modifications and proteolytic breakdown of the protein, and loss of air–blood barrier integrity allows spill-over of pulmonary SP-D into the circulation. For simplicity, only alveolar damage is illustrated. Moreover, only fuzziball SP-D multimers are depicted.

Loss of air–blood barrier integrity is responsible for the outward intravascular leakage of secreted lung proteins and inward edematous flooding in the interstitium and air spaces (274). A concentration gradient of SP-D thus allows SP-D synthesized in the respiratory tract to leak into the bloodstream in acute and chronic lung injury following cigarette smoke exposure, as demonstrated using mice (77, 275, 276) and shown in human subjects (277). Thus, in some settings, including acute cigarette smoke exposure, SP-D may be decreased in BAL while being simultaneously enriched in serum (16, 252, 277). Smoking status is a strong predictor of such translocation (208, 277, 278). Examples include investigations of SP-D variation in COPD (252, 279, 280), asthma (19), and CF (249, 250, 264, 265, 281, 282). Studies in rabbits and humans have provided evidence for molecular size-dependent clearance of proteins from the air spaces of the lung (274); however, although highly anticipated, this study did not conclusively confirmed that LMW SP-D can translocate from the lung into the blood more easily than HMW SP-D (Figure 2). Herbein and Wright (283) reported that SP-D clearance from lavage was elevated in LPS-treated lungs compared with control lungs, due to the increased SP-D uptake in tissue neutrophils, and such clearance may also contribute to decreased alveolar SP-D levels in disease.

Interpretation of the quantitation of SP-D in alveolar fluid and serum in various pulmonary diseases may be hampered because anti-SP-D antibodies may have varying affinities for oxidized and/or crosslinked species, as recently discussed by Atochina-Vasserman (284). HMW and LMW SP-D structural variants have been examined in BAL by native gel electrophoresis in various clinical pulmonary diseases and, as described above, different studies have demonstrated that levels of trimeric SP-D increase in BAL in inflammatory disease, and that trimeric SP-D is composed of both partially degraded, oxidized, and inactive SP-D, as well as a Thr11-glycosylation variant (50 kDa) (18, 19, 242, 249, 252, 262, 267, 285).

### Identification of Modified or Degraded SP-D in Clinical Samples

#### Variation in SNO-SP-D

The presence of SNO-SP-D in BAL from clinically relevant samples has recently been demonstrated. SNO-SP-D was indirectly identified in BAL from patients with Hermansky–Pudlak syndrome type 1 (122) and directly detected in samples from asthmatic patients after segmental challenge with allergen (242). Hence, a role for SNO-SP-D as a disease marker is conceivable, as it is enriched in BAL from patients with pulmonary disease (122, 242). An ELISA-based method to measure circulating SNO-SP-D in COPD patients has been developed using anti-SP-D capture antibodies and antibodies reacting with S-nitrosylated groups for detection; however, this approach did not identify an association with disease severity and found only a weak correlation with radiologist score of emphysema, while possible associations with disease activity have not been verified (286). Nevertheless, detection of circulating SNO-SP-D in additional pulmonary diseases is warranted.

#### Variation in Fucosylated SP-D

A similar ELISA-based approach has been applied for measurement of fucosylated SP-D, demonstrating the presence of a core-fucose in N-glycans groups on serum SP-D, and an association of circulating levels of fucosylated SP-D with COPD outcomes. This modification represents a promising circulating disease-associated SP-D biomarker candidate, although in initial experiments measured levels of fucosylated SP-D could not adequately separate never-smokers, COPD smokers, and COPD (241).

#### Variation in SP-D Degradation Products

An alternative approach to simple SDS-PAGE-based measurement of SP-D degradation products in BAL or serum is permeation chromatography-based size separation; however, both techniques may be biased by genetic size variants (encoded by rs721917) and by oxidative crosslinking. Moreover, enzymatic neutrophil elastase degradation of SP-D and consecutive production of monoclonal antibodies against proteolysis products did not appear to result in immunological recognition of disease-specific SP-D breakdown products (286). Although various proteases are known to degrade SP-D, this approach has not yet been developed sufficiently to enable detection of disease-induced SP-D neoepitopes.

## The Role of SP-D in Respiratory Disease

Circulating SP-D levels and genetic variants in *SFTPD* are associated with the development, progression, and severity of various pulmonary diseases. The variation of constitutional serum SP-D levels spans a >30-fold range (208); therefore, disease-induced serum levels may exhibit considerable overlap with control levels. Nevertheless, the significance of disease-induced levels in prognosis is underscored by association of serum SP-D with mortality in pulmonary disorders, including COPD (287), idiopathic pulmonary fibrosis (288), and ALI/acute respiratory distress syndrome (ARDS) (289). Genetic or phenotypic SP-D variation is associated with ALI/ARDS (289–293), lung injury in critically ill mechanically ventilated patients (294), respiratory distress syndrome (RDS)/bronchopulmonary dysplasia (BPD) (233, 234, 295), community-acquired pneumonia (227, 296), viral infection (297–300), asthma (19, 242), lung cancer (178, 301, 302), pulmonary aspergillosis (281), interstitial lung disease (15, 288, 303–305), and COPD (215, 224, 280). This review is concerned with the role of SP-D in RDS/BPD, asthma, and COPD.

### RDS and BPD

#### Genetic Association

Fetuses carrying the *SFTPD* rs721917 Met11 allele have been associated with spontaneous preterm birth (235), and an initial genetic association of SP-D with pulmonary outcomes in premature infants was suggested by the observations that 2-marker SP-D/SP-A haplotypes including the Met11 allelic SP-D variant were protective against the development of RDS (306) and harmful in BPD (232), respectively. Subsequently, the rs1923537 polymorphism, located downstream of *SFTPD*, was demonstrated as associated with RDS and the requirement for oxygen supplementation at day 28 (a proxy of mild BPD) in very early preterm birth infants (234), whereas other *SFTPD* SNPs, including rs721917 (Met11Thr), were not associated with either RDS or BPD in that study or a subsequent investigation (307). Similar tendencies were observed in another study, although the genetic Met11 SP-D variant appeared to be protective for BPD defined radiologically and with requirement for supplemental oxygen at a gestational age of 36 weeks (295). In the most recent study, several SNPs in *SFTPD*, including the Met11 variant, were found to be positively associated with circulating SP-D levels and harmful in respiratory distress, the requirement for oxygen supplementation at day 28, and respiratory support (233).

Although they generated conflicting data, the above studies support a contribution of SP-D genetic variation to pulmonary outcomes in prematurity, and the discrepancies between studies may be explained by differences in statistical power, different mean gestational ages and the associated variation in requirement for oxygen supplementation at day 28/36, or the investigated SNPs or haplotypes may reflect variation at other, causative variants. No association between *SFTPD* alleles and neonatal mortality has been identified, and this may explain the persistence of high-frequency SNPs associated with respiratory outcomes in prematurity. No association was identified between SP-D SNP variation and diffuse lung disease enriched for genetic surfactant dysfunction (308).

#### Phenotypic Association

Surfactant protein D expression in the fetal distal airways increases with advancing gestation and is evident in 10-week-old fetuses; however, in lungs from infants with RDS and BPD, only open terminal airways were bordered with SP-D expression. Injured areas lined with hyaline membranes, or alveoli filled with hemorrhage, infection, or edema fluid, were lightly stained or unstained, whereas serous cells of bronchial and tracheal glands were consistently stained, particularly in infants with lung inflammation (10).

The percentage of multimeric SP-D in neonatal BAL, which is capable of binding microbial compounds, appeared to be lower in preterm than term infants (309), and alveolar SP-D may essentially be absent in the presence of RDS, increasing after surfactant treatment (310, 311). However, an increase in preterm BAL SP-D the first day (day 1) after birth has been demonstrated (309, 312), in parallel with a transient increase in capillary SP-D levels. This transient, circulatory increase was predominantly observed in infants homozygous for the rs721917 Met11 allele and was more apparent in infants with respiratory distress or receiving respiratory support. Such observations suggest that genetic SP-D variation determines the magnitude of induction of respiratory support-dependent systemic SP-D levels. Serum SP-D levels, which were not significantly affected by antenatal steroids, were positively associated with gestational age, mode of delivery, risk of later septicemia, and risk of respiratory distress (65, 233, 313). A recent study correlated serum SP-D measured at later time points (day 3 and day 7 after birth) in preterm infants and found no relation with the requirement for mechanical ventilation or oxygen, or with the development of BPD using that approach (314).

#### Basic Research

Mouse fetal pulmonary SP-D expression increases with advancing gestation, and levels predominantly elevate shortly before birth, stimulated by vascular endothelial growth factor signaling and glucocorticoid treatment (54, 66–69, 71, 315, 316). Pulmonary SP-D expression is therefore very low in experimental prematurity (317); however, various studies of the *Sftpd^−/−^* lung phenotype do not support a role for SP-D in normal fetal or postnatal lung development, although the vast accumulation of phospholipids in the *Sftpd^−/−^* lung have effects on surfactant homeostasis. Rather, these studies support a role for SP-D in emphysema and fibrosis development during airway remodeling processes later in life, as recently reviewed by Bersani et al. (318).

An early study of baboons showed that both the expression and protein secretion of pulmonary SP-D precede that of SP-A in normal gestational development in the baboon, and they are comparable to, or exceed, adult levels during advancing gestational age. In line with observations of the induction of circulating SP-D with respiratory support, data from premature baboons receiving 100% oxygen for 10 days to produce chronic lung injury indicated that lavage concentrations of SP-A reached a low percentage of that of normal adults, while those of SP-D equaled the amounts present in normal adults. The combined lavage SP-A/SP-D pool reached a low percentage of that of normal adults and the authors concluded that the combined decreased concentration of surfactant host-defense proteins may augment proclivity to infection and worsening injury (319). This suggestion was partly supported by a subsequent experiment demonstrating an increased tendency for lung infection in the same model; however, the major experimental effects were related to massive depletion of SP-A (320). Surprisingly, *Sftpd^−/−^* mice are resistant to hyperoxia, which may be partly explained by phospholipid and SP-B-mediated induction of surfactant resistance to inactivation (321).

Consistent with clinical lung maturation, the major effects of experimental chorioamnionitis are fetal lung inflammation, increased airway surfactants, and increased lung volumes (322–324), while the pro-inflammatory stimulus of chorioamnionitis is also commonly associated with preterm delivery and subsequent RDS (325, 326). A clinical study did not support an association of increased SP-D in amniotic fluid with intra-amniotic infection (327). In contrast, studies of mice and lambs have demonstrated a clear induction of SP-D expression in the fetal lung after LPS treatment (328, 329), although an earlier study indicated greater fluctuations in SP-D expression (330). Furthermore, studies using transgenic mice overexpressing rat SP-D under the SP-C promoter demonstrated that SP-D enhances cytokine production in the fetal and maternal compartments on maternal LPS exposure. Moreover, a significantly higher proportion of the pups born to dams overexpressing SP-D were stillborn after LPS treatment compared with those from wild-type mice (331). Moreover, mice that are doubly deficient in both SP-D and SP-A had delayed parturition and decreased expression of inflammatory and contractile genes (332). A recent study extended these findings, demonstrating that *Sftpd^−/−^* female mice have fertility defects, evidenced by smaller litter size, increased pre-implantation embryo loss, and elevated uterine inflammation, when mated with wild-type males; however, in support of previous findings, maternal LPS administration did not result in increased embryo loss or pro-inflammatory responses in *Sftpd^−/−^* females (333).

In contrast to the surprising association between high intra-amniotic SP-D and preterm birth, addition of recombinant human SP-D to commercial surfactant containing SP-B and SP-C alone improved surfactant function by protecting the premature lung from ventilation-induced inflammation and by increasing its resistance to protein inhibition of surfactant function and changing its biophysical properties and structure when tested in lambs shortly after birth (334). Although pulmonary inflammation was not blocked by SP-D, exogenous SP-D was effective in a model of endotoxin shock in newborn preterm lambs, where intratracheal administration prevented systemic inflammation and decreased cytokine expression in the spleen and liver (169).

### Asthma

#### Genetic Association

Although structural SP-D polymorphisms are not associated with allergic bronchial asthma (220), an association with decreased atopy was identified in black subjects (221).

#### Phenotypic Association

Several studies have documented induction of BAL or systemic SP-D levels in asthma, which may be attributable to a combination of induced SP-D synthesis in airway epithelia (335) and increased air–blood barrier integrity, as described ealier.

Levels of SP-D are increased in BAL samples from allergic asthma patients (336), may further increase after segmental allergen challenge, and be correlated with those of BAL eosinophils, which in turn are correlated with NO content in BAL and oxidized SP-D species (242). Baseline SP-D levels are elevated in serum from patients with allergic asthma, and further elevated after allergen challenge, which is predictive for the late asthmatic response and for eosinophil cationic protein concentrations post-challenge (337).

Recent studies have supported these initial observations and demonstrated that sputum SP-D is increased in severe asthma or severity of exacerbation (338, 339), that serum SP-D increases stepwise in mild to moderate and severe disease, and correlates inversely with lung function and directly with small airway resistance (273). Mackay et al. (19) demonstrated that serum SP-D was increased in severe asthma with mixed eosinophilic and neutrophilic inflammation and enriched for SP-D breakdown products. The latter observation was supported by the recent findings of Fakih et al. (217) of a significantly decreased HMW/LMW serum SP-D ratio in asthmatic patients. In the studies of Mackay et al., the BAL/serum SP-D ratio was reported to be decreased, implicating depletion of SP-D from BAL due to leakage of degraded SP-D to the serum. Furthermore, serum SP-D levels were inversely associated with alveolar neutrophil infiltration and alveolar endotoxin levels (19).

Investigations that did not support a relationship between asthma and SP-D variation include one of the earliest studies of serum SP-D (340), and a recent study of serum SP-D by Akiki et al. (341). These discrepancies may be explained by differences in the distributions of mild and severe asthma between investigations. Serum SP-D enrichment is observed in pulmonary allergies (342–346) and allergic rhinitis (219), which is considered to be a manifestation of the same underlying disease processes as asthma (347), and basic research has demonstrated a link between SP-D and T helper 2 cell (Th2)-mediated inflammation. Thus, differences in clinical observations may also be due to a lack of stratification for different distributions of allergic asthma, or asthma characterized by a Th2-high profile, which accounts for approximately 50% of patients with steroid naïve asthma (348), and the impact of neutrophilic inflammation or pathogenic load, as implicated by the observations of Mackay et al. (19).

#### Basic Research

Allergic asthma models have provided insight into the physiological influence of SP-D in the development of Th2 type allergic asthma. The use of *Sftpd^−/−^* mice as allergic asthma models has provided both clear and subtle allergic asthma phenotypes; however, uniform data have been obtained from studies using exogenous administration of SP-D (110, 197, 349). A large body of studies have used rfhSP-D in treatment protocols and, although rfhSP-D did not efficiently aggregate and opsonize pollen grains relative to native SP-D (92), both SP-D and rfhSP-D appear to dampen the majority of aspects of the allergic phenotype. Intranasal administration of SP-D/rfhSP-D in murine models of pulmonary hypersensitivity induced by diverse allergens and antigens suppresses specific IgE levels in serum, reduces peripheral and pulmonary eosinophilia, and causes T helper 1 cell polarization from the allergic Th2-mediated inflammation to varying degrees (110, 123, 144, 203, 204, 206, 221, 349–353). In one model, beneficial effects of exogenous SP-D were observed when it was administered 6 h after, but not 24 h before, allergen challenge. A single application of rfhSP-D to allergen-sensitized mice led to a dampening of the allergic airway response equilibrium, similar to the effect of budesonide (352).

Moreover, allergen exposure induced SP-D protein levels in an IL-4/IL-13-dependent manner, resulting in increased murine alveolar SP-D levels (353, 354), and this negative feedback loop appears to protect the airways from inflammatory damage after allergen inhalation, as described in a recent review (355). SP-D in lavage and tissue is derived from AT-II cells and Club cells; however, it is also synthesized in hyperplastic goblet cells of inflamed lungs (356).

**Figure 3:**
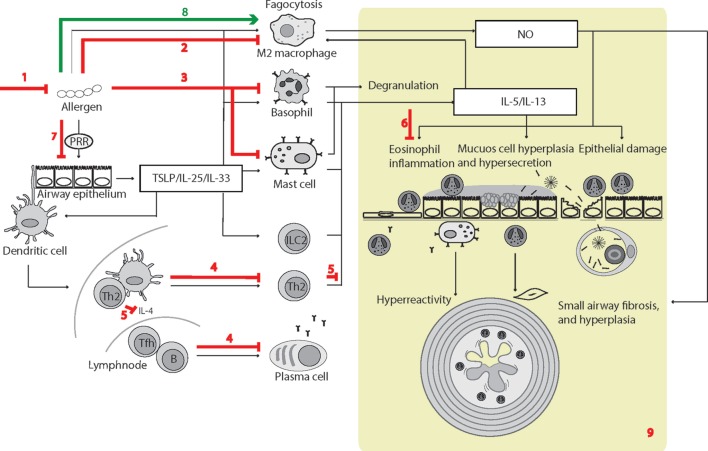
Surfactant protein D (SP-D)-mediated effects in experimental allergic asthma. The overview of cellular functions in allergic asthma was inspired by Lambrecht and Hammad (347) and Fahy (357). The multiple effects of SP-D include (1) removal of allergens by induction of aggregation and accelerating their binding and uptake by alveolar macrophages (92, 154, 358); (2) suppression of M2 macrophage polarization and allergen-stimulated macrophage NO production (123, 352); (3) inhibition of IgE binding to allergens, blocking allergen-induced histamine release by basophils and degranulation by mast cells (153, 154); (4) suppression of peripheral blood mononuclear cell interleukin (IL)-2 secretion (146), lymphocyte proliferation (358), and cytotoxic T-lymphocyte-associated protein 4 (CTLA4)-dependent induction of apoptosis (145). SP-D-mediated T-cell responses are CTLA4 dependent (149); (5) decreased lymphocyte IL-4 and IL-13 release (221); (6) suppression of eosinophil chemotaxis and degranulation, and induction of apoptosis (142, 143); (7) SP-D increases allergen interaction with respiratory epithelium, yet dampens epithelial chemotactic signaling (161); (8) SP-D increases uptake and removal of allergens in macrophages (93); (9) the overall effects of SP-D in allergic asthma *in vivo* include dampening of eosinophilia, alveolar macrophage accumulation, increased specific antibody levels, airway hyperreactivity, subepithelial fibrosis, and mucous metaplasia. These are features, which have either been observed in *Sftpd^−/−^* mice or that are subjected to phenotype rescue by endogenous SP-D, or administration of recombinant SP-D/60-kDa recombinant trimeric fragment of SP-D lacking the N-terminal but retaining a part of the collagen region (110, 123, 144, 203, 204, 206, 221, 349–353, 359). Leakage of pulmonary SP-D to the circulation in allergic asthma has been demonstrated in clinical samples (19). Only trimeric SP-D and fuzziball SP-D multimers are shown for simplicity.

Additional beneficial SP-D effects in asthma may be anticipated in virus-induced asthma, because of the anti-viral effects of SP-D; however, deleterious effects may also be expected due to disease-induced formation of SNO-SP-D. Disease-related proteolysis is suspected to render a fraction of SP-D malfunctioning or deleterious (260), nevertheless, the overall effects of SP-D appear to be beneficial in the context of allergic asthma. The main reported individual steps leading to SP-D-dependent experimental phenotypes are illustrated in Figure 3.

Some of the effects illustrated in Figure 3 remain unclear. SP-D can increase pollen starch granule (PSG)-positive cells *in vitro* and accelerate PSG binding/uptake *in vivo*; however, studies by Winkler et al. (93) demonstrated that it did not affect total clearance of PSGs from the mouse lung nor enhance T-cell proliferation induced by PSG-positive dendritic cells. Hence, the different results obtained using human cell cultures or clinically isolated cells, compared with those from mice models, require further investigation.

A wider role for SP-D in Th2 immunity was recently proposed in a study of infection with the helminth *Nippostrongylus brasiliensis*. Elevated SP-D production in Th2 immunity is partly driven by IL-4 and IL-13. In turn, SP-D can exert negative feedback control of Th2 responses (90, 353); however, whereas allergic asthma studies identified increased type 2 immunity in *Sftpd^−/−^* mice, similar effects were not observed in *N. brasiliensis* infections. The results of these investigations demonstrate that elevated SP-D can enhance type 2 immunity and suggest that SP-D is an important modulator of protective IL-13 producing type 2 innate lymphoid cell-mediated and alveolar macrophage responses against *N. brasiliensis* (90).

### Chronic Obstructive Pulmonary Disease

#### Genetic Associations

Associations of polymorphisms or haplotypes in, or flanking, *SFTPD* with COPD, emphysema, and COPD survival have been identified in various populations and by GWA studies (215, 216, 224–226, 360–362). As reviewed by Lock-Johansson et al. (363), specific SNP associations have not been validated in all investigated populations (363). The SNPs associated with COPD and circulating SP-D levels differed within some investigations, suggesting distinct genetic influences on COPD susceptibility and SP-D levels. In addition, two coding SNPs in *SFTPD* were associated with expiratory lung function in a study of preclinical smoke-induced lung injury (212), suggesting that structural variants of SP-D affect the susceptibility of COPD development in smokers. Recently, Mendelian randomization analyses were used to analyze serum SP-D-associated genetic variants and their association to COPD in the largest study executed until today. Variants were tested for association with COPD risk in 11,157 cases and 36,699 controls and with 11 years decline of lung function in 4,061 individuals. This study concluded that variants associated with increased serum SP-D levels decreased the risk of COPD and slowed the lung function decline (364).

#### Effects of Smoking

The majority of studies of smokers have reported reduced alveolar levels of SP-D (252, 269, 278, 365), and alveolar epithelial injury after LPS instillation was more severe in smokers than non-smokers, with increased circulatory SP-D and decreased BAL SP-D (277). These clinical observations are supported by *in vitro* observations that nicotine can cause reduced levels of SP-D in human airway epithelial cells (366). Additional studies have demonstrated increased circulating SP-D with tobacco smoking or other types of noxious exposure, including data from a twin study showing that smoking monozygotic twins had markedly increased serum SP-D relative to their non-smoking twins, despite the very high heritability of serum SP-D levels (208, 252, 280, 340, 367, 368); however, smoke-induced serum levels may not be clearly evident in mixed populations of respiratory patients, where SP-D levels may be influenced by other processes (369). As described earlier, cigarette smoke disrupts the quaternary structure of SP-D molecules (252). However, an initial attempt to construct an immunoassay for proteolytic SP-D breakdown products in serum did not provide additional information regarding COPD (286).

#### Differentiation between COPD, Smoking, and Other Respiratory Diseases

Whether SP-D levels can differentiate COPD from smoking or other respiratory diseases is uncertain. Some studies have noted significant decreases in SP-D in BAL samples from COPD patients compared with current smokers (252, 279), while others have not (269, 278). In a multicenter study, “Evaluation of COPD Longitudinally to Identify Predictive Surrogate Endpoints” (ECLIPSE), of approximately 2,000 individuals with COPD, higher serum SP-D levels were reported among COPD patients relative to current and former smokers without respiratory obstruction. As the largest difference in serum SP-D levels occurred between non-smokers and current/former smokers, it was concluded that SP-D is a powerful biomarker for smoking; however, there was no difference in serum SP-D levels in individuals with COPD, or in smoker controls with chronic bronchitis, compared with those who did not have this symptom (280). In addition, there are examples of smaller studies that have not shown significant differences between COPD smokers and control smokers (226, 370, 371).

Chronic obstructive pulmonary disease is a heterogeneous disease with several features that overlap with asthma, which has important therapeutic implications for some patients. Data from a randomized control trial of inhaled glucocorticoid therapy in COPD (Groningen Leiden universities chronic obstructive pulmonary disease) confirmed that alterations in airway gene expression may coexist in asthma and COPD, and suggested that Th2 inflammation is important in a subset of patients with COPD who have no history of asthma (372). Despite the vast body of experimental evidence for a role of SP-D in eosinophil-mediated allergy/asthmatic disease, there is no reported evidence linking changes in SP-D expression/function and eosinophil activity in COPD; however, independent studies have provided evidence that supports elevation of circulating SP-D in COPD compared with asthma patients; thus, SP-D may differentiate these two diseases. Yet, these studies may not have controlled sufficiently for the effects of smoking or the severity of asthma (340, 341). Size separation of HMW and LMW serum SP-D appeared to provide a clear distinction between controls and COPD subjects, with the HMW/LMW ratio significantly decreased in COPD after controlling for smoking and additional confounders; however, a similar association was evident for asthmatic patients (217). Additional studies have suggested that smoking-related SP-D variation is prognostic in lung cancer (301, 302).

#### Phenotypic Associations

##### Severity

In the ECLIPSE study, serum SP-D levels were not associated with COPD disease severity, as defined by the Global Initiative for chronic Obstructive lung Disease status, and there was no association with forced expiratory lung function (280). Some smaller studies have reported similar findings (272, 341, 373, 374); however, Ju et al. (375) reported an association with the BODE (Body-mass index, airflow Obstruction, Dyspnea, and Exercise) index of severity (375).

There are contradicting data regarding the association between circulating SP-D and expiratory lung function. The discrepancy indicates that there may be an inverse correlation between serum SP-D and forced expiratory lung function only in smokers with and without COPD (212, 226, 252, 370, 376–379). Such correlations may be difficult to identify in cohorts including both smokers and non-smokers (380), because the two groups exhibit opposite associations with SP-D variation (212). In addition, the correlation may also be affected by differences in COPD treatment.

A recent analysis of data from independent longitudinal population-based cohorts suggested that a large proportion of individuals who develop airflow limitation exhibit a rapid decline in forced expiratory volume in 1 s (FEV_1_) from a normal level of lung function in early adulthood, whereas another fraction had a relatively gradual rate of FEV_1_ decline, but started from a low initial FEV_1_ value (381). In this regard, circulating SP-D has been suggested to provide the means to identify those smokers with low initial lung function who are at highest risk of COPD development and thus eligible for early intervention with pharmacological treatment, in addition to recommendation of tobacco-smoking cessation (212). Nevertheless, longitudinal studies are warranted to validate the use of SP-D measurements as information complementary to spirometric testing for early detection.

##### Treatment

Surfactant protein D is a rather stable marker when measured repeatedly within a few months (252, 280, 382). Nevertheless, Sin et al. (376) reported a significant association between serum SP-D decline and dyspnea improvement within a 3-month study (376). Inhaled corticosteroids (ICS), ICS/long-acting beta(2)-agonist combinations, or prednisolone were also reported to significantly reduce plasma concentrations of SP-D and, in some reports, changes in SP-D levels were associated with symptom scores (279, 280, 373, 383). By contrast, ICS use was independently associated with higher SP-D levels in BAL, and AT-II cells isolated from adult rat lungs responded to dexamethasone treatment by significantly increasing SP-D (279). However, some studies have not validated SP-D as a marker of COPD treatment (374).

##### Activity

Increased levels of circulating SP-D have been reported during exacerbation of COPD compared with stable COPD (384, 385). The observations from these cross-sectional studies were extended by longitudinal studies demonstrating that the level of circulating SP-D decreases in the weeks after hospitalization, due to exacerbation, but ultimately increases some months after hospitalization (375, 386, 387). The variation in levels after the onset of exacerbation appeared to mirror the white blood cell count and was ascribed to the effects of glucocorticoid treatment (386).

Chronic obstructive pulmonary disease subjects from the ECLIPSE cohort, as well as other cohorts, who had the highest serum SP-D concentrations at baseline also had increased risk of exacerbation during the follow-up period (226, 280, 370). The ECLIPSE consortium subsequently performed a more detailed analysis and concluded that the association with SP-D levels did not persist after adjustment for history of exacerbation (388). Overall, the reported data indicate that SP-D is a robust marker for treatment effects, but that the utility of circulating SP-D as marker for prediction of COPD exacerbation may be limited, despite induction of serum SP-D in the acute phase.

##### Emphysema and Mortality

Further data analyses from the ECLIPSE cohort have demonstrated that baseline serum SP-D levels are associated with baseline lung density and its decline over time (389); however, the correlation between SP-D and emphysema was recently challenged by observations of serum SP-D variation in smoking, pulmonary emphysema, and combined pulmonary fibrosis and emphysema (CPFE), where serum SP-D was clearly induced only in the CPFE group compared with the other groups (390). Moreover, a study of indium-exposed workers demonstrated that increased serum SP-D was more closely associated with progression of interstitial changes than with progression of emphysema (391). The ECLIPSE consortium subsequently confirmed that COPD is a highly heterogeneous disease with poor correlations between FEV_1_, symptoms, quality of life, functional outcomes, and biomarkers in general (392), which highlighted the need for patient subgrouping. The consortium then included serum SP-D into a multi-marker cluster analysis of COPD subgroups and found that it was reduced among individuals with more severe emphysema, but higher among those with progressive emphysema. The resulting data suggest that high-circulating SP-D levels could indicate highly active disease and progression, whereas low levels may reflect loss of lung tissue, and thus more severe emphysema. The authors further concluded that the implications of SP-D appeared to differ from cluster to cluster (393). Furthermore, the ECLIPSE and COPDGene multicenter cohort studies both indicated that combinations of multiple biomarkers, including SP-D, are much more strongly predictive of airflow limitation, emphysema, and mortality than any individual biomarker; however, the amount of variance explained by the multiple biomarkers was lower than that of clinical variables (394).

Together with the observation that circulating SP-D is associated with the hard endpoint “all-cause mortality” in COPD (287), its relationship with treatment effects and inverse correlation with FEV_1_ in smokers indicates that SP-D is a useful COPD marker within specified prognostic subgroups and in multi-marker analyses. Multi-marker analyses including SP-D may be useful to limit clinical trials to subgroups of patients likely to benefit from a given intervention or serve as surrogate endpoints.

#### Basic Research

Semi-quantitative studies on aging *Sftpd^−/−^* mice (395) have indicated that they develop progressive emphysema, increasing septal wall thickness (395–397), and subpleural fibrosis (194), which may indicate a phenotype that combines emphysematous and fibrotic changes in the lungs. Highly detailed stereological investigations of the long-term progression of lung parenchymal remodeling, destruction, and septal wall thickening in aging *Sftpd^−/−^* mice were recently undertaken by Schneider et al. (398). These investigations concluded that the unchallenged aging *Sftpd^−/−^* pulmonary phenotype was characterized by airspace enlargement only, without septal wall thickening (fibrosis) or destruction (emphysema). The age-induced airway enlargement was suggested to result from unexplained altered airway mechanics. The authors further suggested that the discrepancies in observations with previous studies might partly result from the different genetic backgrounds of the mouse strains used and ongoing alveolarization in C57BL6 mice (398).

Although the role of SP-D in spontaneous airway remodeling was recently questioned, several studies of *Sftpd^−/−^* mice challenged with noxious stimuli have provided clear evidence for a role of SP-D in pulmonary inflammation and emphysema development, which are essential components of COPD pathophysiology. These results support a role for SP-D in modulating alveolar macrophage activation, oxidant production, and MMP activity, leading to emphysema-like and fibrotic changes of the lung, and histological and morphometric studies have demonstrated airspace enlargement and/or emphysema development associated with *Sftpd* deficiency. Inflammatory and structural changes may be reversed or prevented by local treatment with rfhSP-D or full-length SP-D (200, 202, 399, 400). These changes appear to predominantly affect the alveolar compartment, and there is no apparent SP-D-dependent effect on vascular physiology after cigarette smoke exposure (401).

**Figure 4:**
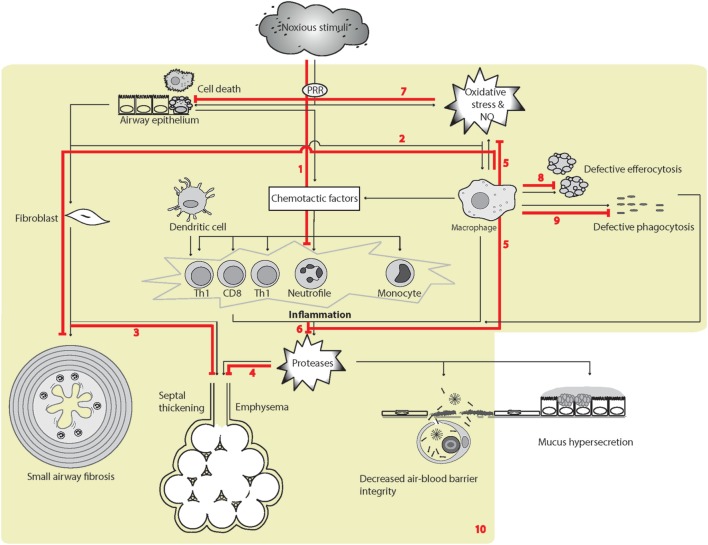
Surfactant protein D (SP-D)-mediated effects in experimental pulmonary inflammation and airspace enlargement in chronic obstructive pulmonary disease (COPD). The overview of cellular functions in COPD was inspired by Barnes (408) and Brusselle et al. (407). Multiple effects of endogenous or exogenous SP-D include (1) suppression of inflammation elicited by noxious stimuli (395, 400, 402, 409). The SP-D-mediated mechanisms includes SP-Ds interaction with immune-regulatory receptors (117, 134, 170, 174, 177), depression of oxidative stress, including iNOS activity, protection of phospholipid oxidation (397, 410–413); (2) decrease of macrophage transforming growth factor-β (TGF-β) production and fibrocyte recruitment (163). These SP-D effects may partly enable suppression of age-induced influences; (3) increased septal wall thickening by fibrotic deposition (194, 397); (4) airspace enlargement and loss of surface area of alveolar epithelia (194, 202, 397, 414); (5) suppression of the production of ROS and NO by macrophages, and possibly additional cell types (125, 194, 395, 409, 411). Fibrotic and emphysematic changes in the lung may also depend on inhibition of iNOS by SP-D (397, 413). (5/6) SP-D suppresses metalloproteinase production in alveolar macrophages (125, 194) and putatively additional cell types. The result is an overall decrease in pulmonary protease activity *via* oxidant-sensitive pathways (125, 194); (7) prolonged alveolar epithelial cell and macrophage survival after cigarette smoke extract exposure or oxidative stress (276, 410); (8) increased efferocytosis (94, 121, 170, 201, 415); (9) opsonization of microbes for phagocytosis (91). (10) The resulting effects of SP-D relevant for COPD-like phenotypes *in vivo* include dampening of chronic low-level pulmonary inflammation predominantly mediated by macrophages and correlated with reduced oxidative stress and protease activity (125, 194), which can prevent changes in pulmonary elastance due to both tissue breakdown and fibrotic build up that occur sequentially with increasing age and exposure to noxious stimuli (395, 397, 409, 413). Leakage of pulmonary SP-D to the circulation in COPD has been demonstrated using clinical samples (280). Only trimeric SP-D and fuzziball SP-D multimers are shown for simplicity.

In contrast to findings in humans with COPD, in *Sftpd^−/−^* mice neutrophil infiltration was not associated with lung remodeling during emphysema development (175). Moreover, alveolar levels of SP-D in mice and rats may increase after pulmonary exposure to noxious stimuli (276, 402–405), in contrast to the majority of findings in humans; however, reductions of SP-D in rat BAL after exposure to noxious stimuli have been reported (406). Viral and bacterial respiratory tract infections amplify the chronic inflammation in COPD by triggering pathogen-recognition receptors, as recently reviewed by Brusselle et al. (407). Additional beneficial SP-D effects in COPD development or progression may be anticipated due to the anti-viral and anti-bacterial effects of SP-D. The main individual steps contributing to the SP-D-depressed experimental phenotypes relevant to COPD are illustrated in Figure 4.

The phenotype of mice deficient for the proposed SP-D receptor, GPR116, is highly similar to that reported for *Sftpd^−/−^* mice, including alveolar enlargement, enhanced ROS production, accumulation of foamy macrophages, and enhanced expression of *Mmp12*. The surfactant lipid changed induced by SP-D may be attributable to its effects on alveolar type II cell activity (177, 193, 414). Although GPR116 deficiency also causes an emphysema-like phenotype that is associated with alveolar macrophage activation (177, 416), the phenotypes of neither GPR116 nor *Sftpd*-deficient mice fully reflect those of human COPD. Mouse alveolar macrophages become foamy with lipid-laden phagosomes due to extensive uptake of surfactant lipids (177, 417, 418); however, in human pathology, foamy macrophages are found in diverse disorders, including diffuse pan-bronchiolitis or bronchiolitis associated with bronchiecstasis (419), diseases of surfactant homeostasis (420), extrinsic allergic alveolitis (421), and tuberculosis, rather than in COPD. Moreover, the alveolar accumulation of surfactant lipids in the two types of gene deficiency appears to reflect aspects of pulmonary alveolar lipoproteinosis, derived from defective granulocyte macrophage colony-stimulating factor signaling (124, 396, 422), or other types of macrophage exhaustion. Thus, the appearance of foamy macrophages in the *Sftpd^−/−^* lung may not be specific to COPD-related processes, although induction of MMP-12-synthesizing foamy macrophages results after exposure of mice to cigarette smoke (276), and foamy macrophages provide a source of ROS and inflammatory signaling in mouse models as a result of *Sftpd* deficiency (125).

## SP-D in Non-Respiratory Diseases

Genetic SP-D variation, altered local protein expression, and serum variation have been reported in extrapulmonary diseases involving autoimmune disorders [including rheumatoid disease (213, 423–426) and diabetes (229, 427)] and in diseases involving specific organs [including the intestines (230, 428), skin (20), brain (41), and large arteries (214, 429)]. In some cases, the association between circulatory SP-D and extrapulmonary disease may be partly explained by the coexistence of respiratory disease or the pulmonary effects of disease (430–434). The antimicrobial or anti-inflammatory effects of SP-D at extrapulmonary sites have been demonstrated using animal models or cell culture, including disease models of the eye (258, 261, 435), pancreas (436), kidney/urinary tract (437, 438), gastric mucosa (439–441), intestine (442), gestational tissue (27, 331, 443), and large arteries (48). Inflammation-modulatory effects of SP-D at extrapulmonary sites is, to a certain degree, similar to SP-D-mediated effects in the pulmonary compartment, yet unique extrapulmonary effects are emerging in studies of CVD, such as atherosclerosis, and also in metabolic disease.

### Cardiovascular Disease

#### Association with All-Cause Mortality

A recent study of constitutional circulating SP-D measurements in an elderly twin population, where subclinical disease was likely to be present, demonstrated a relationship between the highest SP-D levels and increased all-cause mortality. Female twins with the highest SP-D levels had a significantly increased risk of dying before their co-twin during the study follow-up period. Adjustment of this analysis for intrapair differences in smoking pack-years did not affect the association, indicating that SP-D is not merely a proxy for smoking in reflecting mortality (444). A study of dementia in the elderly reached a similar conclusion about the association with all-cause mortality, without a gender-bias (445). Hence, it is possible that SP-D is associated with CVD, which is the number one cause of death globally.^1^

#### The Relationship between Pulmonary Impairment and CVD

The sources of circulating SP-D are not entirely clear, and it is possible that spill-over of SP-D from the diseased arterial wall into the circulation may affect total serum levels, in addition to the lung, as a main contributor. COPD and coronary artery disease (CAD) frequently coexist, COPD is an independent risk factor for CAD and increased risk for cardiovascular mortality, and patients with diagnosed and treated COPD are at increased risk for hospitalization and death due to CVD (432, 446–451). These correlations suggest that the association of SP-D with all-cause mortality may be rather complex. Some recent studies have demonstrated that SP-D levels correlate with alveolar leakage in heart failure (433), and with the presence of submassive pulmonary embolism (434), supporting the hypothesis that variation in circulatory SP-D results from disease-mediated lung damage in some types of CVD.

#### Genetic Association

Recently obtained data indicated nominal associations between *SFTPD* coding variants, including the Met11Thr polymorphism, and subclinical atherosclerosis, and demonstrated that the direction of the effects was dependent on tobacco smoking, but independent of circulating SP-D levels (214). An additional study identified a borderline significant association of the Met11Thr polymorphism with advanced atherosclerosis, and this variation was observed to significantly contribute to the risk of arterial stenosis when included in a complex model with other inflammatory gene variants (222).

#### Phenotypic Association

As suggested above, innate immune factors, including SP-D, may provide a link between respiratory and vascular morbidity and mortality. In this context, circulating SP-D was positively associated with both all-cause mortality and cardiovascular mortality in a large study of the Vancouver Coronary Angiography Cohort by Hill et al. (429), and inclusion of SP-D into a multi-marker analysis improved risk prediction across all levels of risk. There was also a significant positive association of SP-D with triple vessel disease and a borderline association with total cholesterol; however, no associations with other serum lipid fractions or diabetes mellitus were observed in that study. SP-D was also associated with all-cause mortality in the ECLIPSE COPD cohort (287), as described above, and in a study of the relationships among COPD, coronary artery calcification, and mortality using the same cohort, circulatory SP-D was correlated with Agatston score (432). The relationship between SP-D and CVD is further supported by the observation of Hu et al. (452) of positive associations between circulatory SP-D levels and carotid artery intima-media thickness and severe coronary artery calcification, which persisted after multivariate adjustment. Hill et al. (429) and Hu et al. (452) did not report BAL SP-D or other means of monitoring the presence of lung disease and, therefore, did not address the question of the organ source of circulatory SP-D.

#### Basic Research into the Role of SP-D in Atherosclerosis

Surfactant protein D expression, as well as expression of the SP-D-receptor, OSCAR, which induces secretion of TNF-α by CCR2 + inflammatory monocytes exposed to SP-D, was localized to the tunica intima and tunica media of clinical atherosclerotic plaques (48, 133). Arterial SP-D expression is documented both in endothelial cells (48) and coronary smooth muscle cells (47), where the pattern of expression regulation implicates SP-D in modulation of inflammation, and TNF-α induces SP-D expression in both cell types (47, 63). Direct interaction between LMW, but not HMW, SP-D and HDL, LDL, and oxLDL was demonstrated using ELISA-based assays (36). These observations suggest that the dominant effects of SP-D in the vasculature may not be the multivalent interactions with microbes, but rather may include specialized LMW SP-D-mediated effects on lipid metabolism.

The SP-D receptor, OSCAR, is expressed by cell types relevant for atherogenesis, including monocytic cells (133), neutrophils (453), dendritic cells (454, 455), and endothelial cells (456). *Sftpd* deficiency is shown to be protective in a diet-induced atherosclerosis model in C57BL/6 mice. Endogenous SP-D can influence systemic plasma lipids and inflammation, reducing HDL cholesterol and triglyceride levels while increasing those of TNF-α in mice maintained long term on a cholate-rich diet. Intravenous treatment with rfhSP-D for 5 days resulted in reduction in HDL cholesterol, but also, in parallel, in LDL cholesterol and total cholesterol, while there was no effect on triglycerides. The role of SP-D-mediated modulation of plasma lipids in atherogenesis is thus uncertain. However, the long-term effects of these events were not studied (48). A subsequent study using double-deficient apolipoprotein E (*Apoe*)*^−/−^Sftpd^−/−^* mice showed similar quantitative effects in decreasing lesion size. Moreover, *Sftpd* deficiency resulted in less macrophage accumulation, and more smooth muscle cell coverage of lesions (457). In that study, endogenous SP-D reduced several plasma lipid fractions, including total cholesterol and induced circulatory IL-6, rather than TNF-α. In contrast to the two previous studies, a study using double-deficient LDL-receptor (*Ldlr*)*^−/−^Sftpd^−/−^* mice did not provide clear evidence for a role of SP-D in atherogenesis (Sorensen, unpublished data).

Bridges et al. (410) reported that SP-D was a potent inhibitor of oxidation of LDL to oxLDL in *in vitro* assays, but later ascribed the majority of the effects to residual EDTA in the SP-D vehicle in a published *erratum*. However, the consequences of *Sftpd* deficiency in atherogenesis may also result from the antioxidant effects of SP-D and the effects on the formation of ROS and in NO-mediated signaling, although these aspects have not been investigated in detail in the vascular system.

A potential link between low SP-D levels and the sequela of atherosclerosis was recently demonstrated using cerebrospinal fluid samples from patients with cerebral infarction (41). Almost simultaneously, a mouse model of cerebral ischemia was analyzed, but showed no effect of *Sftpd* deficiency; however, that model was based on permanent occlusion of the middle cerebral artery and did not involve atherosclerosis (49).

**Figure 5:**
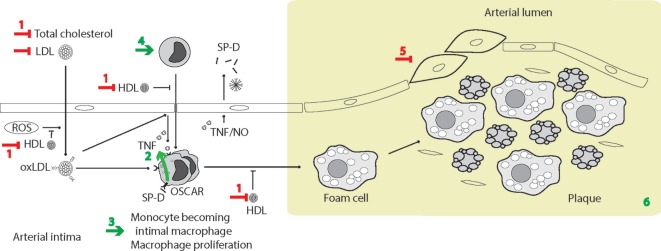
Surfactant protein D (SP-D)-mediated effects in atherogenesis. The simplified overview of cellular functions in atherogenesis was inspired by Skaggs et al. (458). Effects of SP-D in a long-term diet-induced model of atherosclerosis include (1) repression of high-density lipoprotein (HDL)-cholesterol levels (48) and also additional plasma lipid levels in a genetic model (457); (2) model-dependent induction of tumor necrosis factor-α (TNF-α) *in vivo* (48, 457) and TNF-α induction in monocytes *in vitro*, dependent on osteoclast-associated receptor (OSCAR) signaling (133); (3) induction of macrophage proliferation (457); (4) induction of circulating monocytes; (5) decreased coverage of smooth muscle in plaques (457); (6) the result is an overall increase in atherosclerotic plaque formation with accumulation of foam cells and cholesterol crystals, accompanied by disturbed plasma lipid levels in *Sftpd^−/−^* mice compared with *Sftpd^+/+^* mice (48, 457). Moreover, TNF-α and NO increase endothelial SP-D expression (63) and circulating SP-D is increased in clinical cardiovascular disease (CVD) (25, 429). Only trimeric SP-D and fuzziball SP-D multimers are shown for simplicity.

The main reported individual steps leading to SP-D-enhanced experimental outcomes relevant for atherosclerosis are illustrated in Figure 5.

### Metabolic Disease

#### Relationship between Pulmonary Impairment and Metabolic Disease

In addition to the association of SP-D with CVD, possible causes of the association between circulating SP-D and all-cause mortality may include the role of SP-D in metabolic homeostasis. As described earlier, the brain, pancreas, gut, and endothelium express SP-D, and these locations suggest potential effects in systemic metabolism. Diabetes and metabolic syndrome are frequent comorbidities in COPD patients (459–462). COPD, diabetes, and the metabolic syndrome are all related to systemic inflammation, which may explain their cooccurrence (463). Moreover, pulmonary function impairment is well recognized in diabetes and may be attributable to increased pulmonary bacterial colonization due to hyperglycemia and exacerbation of COPD, among the additional causes recently discussed by Kinney et al. (464). By contrast, a reduced ability to eradicate pathogens may cause frequent respiratory tract infections, and chronic inflammation resulting in insulin resistance and type 2 diabetes, as reviewed by Fernández-Real and Pickup (465). Moreover, cigarette smoking is an independent predictor of type 2 diabetes, although the link between lower vital capacity and diabetes risk is independent of cigarette exposure and stronger in never-smokers (466).

Obesity is the most frequent cause of insulin resistance (467) and, although impaired lung function is present before the development of metabolic syndrome or diabetes, the associations between pulmonary and metabolic impairment may, at least partly, result from the effects of obesity, as recently shown in the longitudinal Strong Heart Study (468). Multiple cellular and molecular mechanisms of the innate immune system may contribute to this relationship, as reviewed by Lackey and Olefsky (469). Several studies have independently identified positive associations between lung function impairment and full metabolic syndrome or obesity (470–473), and metabolic biomarkers are predictors of impaired lung function in response to particulate inhalation (474).

Overall, the close relationship between metabolic and respiratory impairment suggests that lung innate immunity, as inferred from genetic or phenotypic SP-D variation, could reside at the intersect of inflammation, obesity, and insulin resistance.

#### Genetic Association

Multivariate genetic analysis was used to investigate genetic associations of serum SP-D with factors involved in metabolic syndrome in a twin study (GEMINAKAR). The study found that genetic correlations between traits differed, but were close to zero for SP-D versus body mass index (BMI), which may indicate that the common genetic contribution to the regulation of the traits (i.e., pleiotropy) is significant but limited. There was no significant genetic correlation between serum SP-D and HDL cholesterol or total cholesterol, and the study did not investigate possible genetic associations between SP-D and diabetes (208). A subsequent study by Ortega et al. (51) demonstrated that a common SNP variant in the *SFTPD* promotor, which is positively associated with circulating SP-D levels, was enriched in individuals with lower BMI and decreased percentage fat mass. A study by Pueyo et al. (229) showed that the presence of the variant SP-D allele, Met11, increased the odds of developing insulin resistance and type 2 diabetes, especially among women. Moreover, this SNP, and others in the *SFTPD* genomic region, were significantly associated with glucose homeostasis, insulin sensitivity, and type 2 diabetes, according to GWA datasets, and these associations were independent of circulating SP-D concentrations (229). A borderline significant inverse association was observed between free LPS concentration in plasma and circulating SP-D in subjects homozygous for Thr11, but not those homozygous for Met11, in a small subpopulation of the study. This observation indicates that the different genotypes may predispose to differing buffering capacity in metabolic endotoxemia (229), consistent with observations by Leth-Larsen et al. (37), showing that Thr11 SP-D bound preferentially to LPS compared with Met11 SP-D (37).

#### Phenotypic Association

Fernández-Real et al. (427) evaluated serum SP-D in four different cohorts and found that SP-D concentrations were significantly decreased in non-obese populations with type 2 diabetes, and that they were correlated with several metabolic variables, including BMI, and fasting and post-load glucose levels. Obese subjects showed significantly decreased serum SP-D concentrations, and weight loss led to further significantly decreased serum SP-D concentrations, while serum SP-D concentrations were not significantly associated with lung function parameters in these studies. When multiple linear regressions were performed using data from subjects with altered glucose tolerance, fasting glucose and fasting triglycerides were identified as independently contributing to 33% of SP-D variance (427). However, a recent study by López-Cano et al. (475) demonstrated increased serum SP-D in diabetes in obese patients with type 2 diabetes. Moreover, serum SP-D in this population was independently and inversely associated with FEV_1_ in the obese diabetes patients and the authors concluded serum SP-D might be a useful biomarker for detecting lung impairment in obese patients with type 2 diabetes.

The findings of Ortega et al. (51) supported previous observations that *SFTPD* was expressed in human adipose tissue and adipocytes, and this expression was decreased in both omental and subcutaneous adipose tissue from obese subjects with and without type 2 diabetes, compared with the control group. Omental SP-D expression was inversely associated with several metabolic variables, including BMI, percentage fat mass, waist circumference, and fasting glucose; and positively associated with the expression of insulin receptor substrate 1 and fatty acid synthase, suggesting that SP-D may effect glucose tolerance in fat tissue or *vice versa*.

At least two previous studies demonstrated decreased constitutional circulating SP-D in obese or overweight subjects after correction for gender, smoking, and age, which may support an initial influence of the processes leading to accumulation of fat tissues on the correlations between SP-D and metabolic impairment. As a part of the Danish population-based twin study on metabolic syndrome, GEMINAKAR, serum SP-D was identified as significantly and inversely associated with weight and waist circumference in men, and with BMI in both sexes (476). Similar findings were obtained from a Chinese population-based cohort (271). Moreover, the relationship was also observed in patients who were obese and had ALI (477).

#### Basic Research into the Role of SP-D in Metabolism

Three independent studies have demonstrated a link between *Sftpd* deficiency and obesity (457, 476, 478). According to a study by Stidsen et al. (478), body mass development in *Sftpd*-deficient mice was significantly increased compared with that of corresponding wild-type mice receiving both *ad libitum* and fixed energy intake diets. The study also identified a significant redistribution of body fat in parallel to obesity-related insulin resistance, as well as increased metabolic endotoxemia associated with the *Sftpd* deficiency in this model (478). Relative insulin resistance in *Sftpd*-deficient mice was later validated by Hirano et al. (457). Additional links between SP-D and body fat mass include the observation of decreased SP-D expression in human adipose tissue with increasing BMI (51), and direct SP-D interactions with fatty acids (479).

Furthermore, SP-D was linked to metabolic homeostasis in two independent studies seeking to identify genes involved in β cell adaptation during mouse pregnancy or in rat perinatal regenerating pancreas. The studies demonstrated that *Sftpd* mRNA and protein levels in β cells exhibited large but transient perinatal increases (480, 481). TNF-α- and IL-1β-induced expression of SP-D in islets was demonstrated (480), suggesting that TNF-α induction of SP-D is a general effect for many cell types.

No direct role of SP-D in experimental diabetes development has been demonstrated; however, the development of diabetes in mother rats is accompanied by delayed pulmonary histogenesis and decreased SP-D expression in the pups (482). Moreover, viral neutralization by SP-D *in vitro* was abolished in the presence of glucose at levels commonly found in diabetic mice (483), which may contribute to reduced anti-viral protection in diabetes.

## Summary, Including Unresolved Questions

### SP-D Effects and Variants

Widespread SP-D expression has been documented in a multitude of studies and underscores the fact that, although the lungs remain a major site of SP-D synthesis, the essential effects of SP-D in innate immunity can be expected at numerous additional sites, and various examples of such extrapulmonary effects have now been investigated and documented. For many years, research into SP-D-mediated immune-modulation has primarily focused on its roles in phagocytosis and efferocytosis, opsonization, and oxidative signaling in phagocytes. However, analyses of SP-D interactions with various white blood cells, epithelial cells, fibrocytes, and smooth muscle cells have provided more detailed insights into SP-D-modulated pathologies, and imply that additional cellular SP-D-mediated effects remain unexplored. Further examples include endothelial cells and adipocytes, where SP-D expression is reported, but where the effects of its expression remain largely unknown. Gaps in knowledge regarding the role of SP-D in non-immune cells and the levels of expression of SP-D during homeostasis and disease have the potential to hamper the design and interpretation of both *in vivo* experiments and clinical biomarker studies.

In support of observations of SP-D-induced cellular signaling, more than 10 different SP-D receptors or receptor complexes have been identified; however, the majority of studies suggesting new SP-D receptors have yet to be validated by independent researchers. In the majority of reports, the pulmonary effect of SP-D signaling is the dampening of inflammation. Pulmonary suppression of inflammation is thus the documented outcome when applying SP-D or rfhSP-D treatment, despite the potential inflammatory side effects of SP-D variants, which are unable to form HMW SP-D due to modifications, or which may represent proteolytic breakdown products.

Low-molecular weight SP-D is characterized by preferential binding to LPS and circulating lipoproteins, compared with HMW SP-D; however, the effects on pro- or anti-inflammatory cellular signaling resulting from the recognition of such complexes have not been explored. Moreover, it is unknown to what degree interaction of SP-D with LPS and lipoproteins mediates assembly of LMW into HMW forms *in vivo*, as observed with immobilized ManNAc ligands *in vitro*. However, observations suggest different effects of LMW SP-D compared with HMW SP-D and studies of a previously proposed role for collectins in quenching the immune-activating effects of hydrophobic polymers, such as lipids or cholesterols from lipoprotein particles (484), are warranted, particularly for LMW SP-D.

In contrast to the primarily anti-inflammatory effect of SP-D in the lung, during development of mouse atherosclerosis the major impact of SP-D appears to be enhancement of inflammation (48, 457) and this is also the case during fetoplacental development (331). Such observations could potentially be explained by site-specific expression of SP-D receptors. Determination of the contribution of SP-D-mediated OSCAR signaling to respiratory and non-respiratory disease may help to clarify the reasons underlying conflicting observations of pro- and anti-inflammatory effects of SP-D.

There is a paucity of *in vivo* data confirming native SP-D structural size variation-dependent effects in disease, although initial attempts have been made using *Sftpd^−/−^* mice overexpressing the common human *SFTPD* Met11Thr variants to determine their effects on the degree of SP-D multimerization. By contrast, various studies have demonstrated that treatment with the LMW rfhSP-D molecule may rescue histogenesis in *Sftpd^−/−^* mice and resolve inflammation both in *Sftpd^−/−^* and *Sftpd^+/+^* mice. This suggests that this particular fragment may be capable of signaling through the same set of cellular receptors as endogenous SP-D, or that some *in vivo* inflammatory effects may be ameliorated by high local concentrations of SP-D, regardless of the distribution of HMW and LMW SP-D. However, rfhSP-D has also failed to demonstrate effects in several settings and the question of the extent to which the effects of full-length multimeric SP-D may be mimicked by high levels of trimeric SP-D has yet to be fully answered.

The degree to which *SFTPD* variants affect SP-D structural size in disease also remains to be elucidated. Nevertheless, the Met11Thr polymorphism, which affects the degree of multimerization in non-diseased populations, has also been identified as associated with numerous respiratory diseases, as well as with CVD and metabolic diseases. Such *SFTPD* Met11Thr associations with disease may indicate that, not only the expressional levels of SP-D but also its ability to form multimers, are essential SP-D-mediated effects beyond interaction with multivalent ligands and clearance thereof. Overall, the majority of data from genetic studies of this particular polymorphism indicate associations of the Thr11 allele with diverse clinical conditions. However, this allele has also been reported as protective in some diseases, including severe respiratory syncytial virus infection, which can cause infant death, and in which the allele is observed at a relatively high frequency.

### Phenotypic SP-D Variation Induced by Disease-Related Processes

The lungs are among the five largest organs of the human body, and SP-D concentrations in the alveolar lining fluid appear to be higher relative to circulating levels. Thus, loss of air–blood barrier integrity in respiratory disease may cause pulmonary SP-D to translocate, leading to an increase in systemic levels, despite the huge variation in constitutional levels. Clinical SP-D deficiency is not documented; however, BAL levels of SP-D may decrease during respiratory disease to a level that does not allow immunodetection, while circulatory levels rise. However, it has not been conclusively confirmed that LMW SP-D will translocate from the lung into blood more easily than HMW SP-D, and just two studies (19, 217) have confirmed increased levels of circulating LMW SP-D in respiratory disease.

The translocation of SP-D appears to be reflected in a change of the ratio between BAL SP-D and circulating SP-D. This ratio is effective for differentiation of respiratory cases from controls and smokers, which is not always possible using isolated serum or BAL measurements (252); however, due to the invasive nature of sampling, SP-D measurements in BAL do not provide useful means for disease monitoring. Few attempts to monitor LMW SP-D, or breakdown products of SP-D, have been reported, and these primarily rely on the use of native gel electrophoresis, which may not be applicable to large-scale analysis. The development of immunoassay-based methods, which have previously been used to detect circulating extracellular matrix epitopes (485, 486), is warranted for studies of SP-D as disease biomarker. Whether particular LMW SP-D species are enriched in the lung or other organs requires further exploration.

### Biomarker Utility

This review has focused on SP-D variation as prognostic marker for infant respiratory distress syndrome and BPD, moderate and severe asthma, and COPD. The involvement of SP-D in these clinical respiratory diseases is partly supported by genetic analyses. The data regarding *SFTPD* associations with RDS and BPD are conflicting, and an association of *SFTPD* with atopy has been demonstrated, but not with asthma. Moreover, associations of polymorphisms within or flanking *SFTPD* have been identified in various populations of COPD patients and by GWA, although the overlap between observations is not complete.

By contrast, circulating SP-D levels appear to be uniformly increased with disease severity, particularly in RDS and asthma, and are associated with further progression or exacerbation of disease, and with mortality from COPD. Clinical measurements of SP-D primarily support that the total circulating SP-D can be used to correct for the background variation in SP-D in interstitial lung diseases or to facilitate differentiation of idiopathic pulmonary fibrosis from other idiopathic interstitial pneumonias when included into a multi-marker array (15, 304, 487). Nevertheless, measurement of total circulating SP-D may also prove to be valuable in multi-marker-based identification of disease subtypes for pharmacological testing in other types of respiratory disease, including COPD (393).

#### RDS and BPD

Important information has been obtained about SP-D variation in premature infants, including the normal range of SP-D in term and preterm newborns and the influence of perinatal factors; however, there is a paucity of independent studies validating the observed association between the highest serum SP-D levels measured day 1 after birth and the most severe outcomes in premature infants, which warrants further validation.

#### Allergic Asthma

Recent research findings support increased circulating SP-D in moderate or severe asthma and in addition to allergic rhinitis. Yet, SP-D breakdown products were also enriched in the blood system in severe asthma, indicating that such fragments may provide a more specific measure of disease activity.

#### Chronic Obstructive Pulmonary Disease

Although SP-D levels are highly increased in response to smoking, the highest circulating SP-D levels appear to be not only proxies for smoking but also related to disease outcomes after adjustment for smoking, hence providing a robust marker for treatment effects. Association with mortality has been identified in CVD in cohorts selected, and not selected, for COPD. The presence of CVD is thus anticipated to be a significant contributor to induction of circulating SP-D levels in COPD. The authors of the ECLIPSE study stressed that division of the COPD population into more homogenous subgroups with similar prognoses provides the potential to test interventions. That study further underscored that monitoring of biomarker arrays including SP-D is useful as an indicator of disease prognosis, or for prediction and monitoring of clinical responses to an intervention; however, this strategy is yet to be validated in randomized clinical trials.

In general, there appears to be an untapped potential for monitoring circulating LMW SP-D or SP-D neoepitopes, with the aim of separating disease cases from controls, as well as for use in disease prognosis. Specific proteases are known to degrade SP-D, and this aspect has not yet been pursued sufficiently to identify disease-induced SP-D neoepitopes. More elaborate immunoassay-based methods, which have been previously used to detect circulating extracellular matrix epitopes, are warranted for studies of SP-D breakdown product variation in respiratory, as well as non-respiratory, diseases and experimental data support the presence of such circulating breakdown products. This approach has the potential to provide measures of disease and organ specific SP-D breakdown products.

It remains to be determined whether those subjects presenting with the highest circulating SP-D levels are prone to the development of clinical disease due to the presence of preclinical lung injury or CVD and if monitoring of SP-D may facilitate early intervention.

Circulating SP-D is confounded by adiposity and diabetes, and experimental and genetic links have been established to these disorders; however, these associations have not been explained mechanistically. The correlation between circulating SP-D and adiposity is inverse and may, therefore, counteract phenotypic associations with both respiratory disease and CVD.

## Perspectives: Recombinant SP-D Treatment

Alveolar levels of endogenous SP-D may be vastly decreased in respiratory disease, particularly RDS, but also in severe asthma and COPD, and preclinical tests of treatment by local SP-D enrichment have been undertaken in animal models. Alveolar SP-D may be further modified, cleaved, or tightly bound to other components.

### RDS and BPD

Pulmonary instillation of SP-D is beneficial, for example, in preterm ventilated lambs representing a clinically relevant model for preterm surfactant needs, and also in allergic asthma models and smoke-induced pulmonary injury. While currently used surfactant therapeutic formulations lacking SP-D have greatly reduced mortality from RDS, a significant proportion of patients develop oxygen dependency and ventilatory requirements extending beyond the first week of life, especially among survivors of very low-birth weight and extreme prematurity. Using the preterm lamb model, treatment effects were more pronounced in animals treated with surfactant (Survanta^®^) enriched with SP-D versus those receiving surfactant alone. This suggests that there is a therapeutic potential for the use of SP-D as a supplement to current surfactant formulations for the treatment of neonatal RDS or development of BPD, which merits clinical investigation.

### Allergic Asthma

A multitude of *in vivo* data support the beneficial effects of exogenous SP-D treatment for allergic asthma and pulmonary infections. Treatment with exogenous SP-D may have dual effects in asthma, targeting both inflammation and fibrotic development, and induced anti-inflammatory effects comparable with those obtained by treatment with budesonide. By contrast, Survanta^®^ did not have a suppressive effect in a mouse model of a house dust mite-induced inflammatory asthma process. In addition, exogenous SP-D could reduce fibrotic depositions in house dust mite allergen challenged mice, or when administered throughout the experimental period of bleomycin treatment for the induction of pulmonary fibrosis. However, it remains to be seen whether exogenous administration of SP-D can attenuate fibrosis in wild-type mice and when it is administered only during the fibrotic phase. Such experiments will be crucial to add weight to the potential of SP-D as a therapeutic intervention in allergic asthma or other respiratory diseases characterized by fibrotic deposition, including BPD, COPD, and idiopathic pulmonary fibrosis.

### COPD and Atherosclerosis

The effects of exogenous SP-D in models of COPD and atherosclerosis require validation by independent laboratories and further mechanistic studies. However, observations from basic cardiovascular studies and studies of metabolism, suggest that systemic side effects from exogenous SP-D treatment include disturbance of circulating lipoprotein levels and whole-body fat distribution. Such observations imply that local administration of SP-D for the treatment of respiratory disease may be preferred over systemic induction of SP-D expression. However, the understanding that SP-D may also be harmful in atherogenesis and in infection during fetoplacental development suggests that the development of pharmaceutical means to induce tissue or cell type-specific inhibition of SP-D is warranted.

### Additional Potential Types of Treatment and Applications

Additional potential treatments that have been suggested include the use of covalently bound complexes, comprising antibody crosslinked to point mutated SP-D (299, 488), or introduction of specific N-linked glycans (489), in enhanced anti-viral or anti-bacterial therapies (490); for example, genetically engineered SP-D, which is resistant to proteolysis and relevant for the treatment of *P. aeruginosa* (250, 260); reduction of lung inflammation and injury after allogeneic hematopoietic stem cell transplantation (491); inclusion of SP-D in nasal spray for chronic rhinosinusitis with polyps (13); alleviation of pneumonia severity and intestinal injury in *Staphylococcus aureus* pneumonia (441) or pulmonary aspergillosis (205). Moreover, therapeutic approaches have been suggested to increase transcription of SP-D (62, 492).

### Development of Therapies

No clinical trials treating patients with recombinant SP-D have yet been undertaken, despite the body of preclinical data supporting significant treatment effects. Nevertheless, the early observation that recombinant SP-D prevented endotoxin shock in preterm lambs paved the way for the formation of a spin-out company from Cincinnati Children’s Hospital Medical Center, “Airway Therapeutics”^2^ in 2011, with the goal of producing full-length recombinant SP-D as a replacement protein therapy for the prevention of BPD in premature infants, and this product has received orphan designation status for BPD in the US and European Union. According to the website, weare.techohio.ohio.gov, AT-100 is currently in an advanced preclinical development stage. The GMP-production process is in development and investigational new drug-enabling preclinical experiments are being conducted.

Although airway therapeutics has focused on producing full-length SP-D and may be close to conducting trials, an alternative approach would be to produce rfhSP-D, which can be synthesized by *Escherichia coli*, and an rfhSP-D-based treatment has secured funding for continued development (Professor Howard Clark, University of Southampton and Honorary Consultant in Pediatrics at the Southampton University Hospitals Trust, personal communication).

In addition to the intellectual property rights for production of full-length SP-D or rfhSP-D for treatment and prophylaxis of respiratory disease (493, 494), more patents have been obtained to facilitate the commercial development of SP-D-based monitoring of respiratory disease or pharmaceutical treatments based on recombinant SP-D, or fragments thereof. A non-exhaustive list of patents and patent applications cover SP-D as marker for dyspnea (495), as a marker of steroid response in asthma and COPD (496), glycyrrhetinic acid for reduction of SP-D expression (497), SP-D modulation of eosinophil activity (498), combined administration with lysozyme and SP-D in respiratory prophylaxis (499), and recombinant chimeric SP-D-antibody-fragment proteins for enhanced neutrophilic uptake of pathogens (500).

The critical step for the future development of SP-D-based therapy will be the outcomes of well-performed clinical trials confirming the hypothesis that exogenous SP-D will improve neonatal RDS or BPD. The results of such trials have the potential to facilitate further development or testing of SP-D treatment in additional diverse settings, including fibrosis of the lung, allergic asthma, and COPD.

## Author Contributions

GS conceived, drafted, and approved the final version of the manuscript and is accountable for all aspects of the work.

## Conflict of Interest Statement

The author declares that the research was conducted in the absence of any commercial or financial relationships that could be construed as a potential conflict of interest.

## Footnotes

^1^www.who.int.

^2^http://www.airwaytherapeutics.com/about-us/.

## Abbreviations

AHR, airway hyperreactivity; ALI, acute lung injury; ARDS, acute respiratory distress syndrome; AT-II cells, type II alveolar cells; BAL, bronchoalveolar lavage; BMI, body mass index; BPD, bronchopulmonary dysplasia; CAD, coronary artery disease; CCR2, C-C chemokine receptor type 2; CD14, cluster of differentiation 14; CF, cystic fibrosis; CL-10, collectin-10; CL-11, collectin-11; CL-12, collectin-12; COPD, chronic obstructive pulmonary disease; CTLA4, cytotoxic T-lymphocyte-associated protein 4; CTLD, C-type lectin domain; CVD, cardiovascular disease; DC-SIGN, dendritic cell-specific intercellular adhesion molecule-3-grabbing non-integrin; ECLIPSE, evaluation of COPD longitudinally to identify predictive surrogate endpoints; EGF, epidermal growth factor; EGFR, epidermal growth factor receptor; ELISA, enzyme-linked immunosorbent assay; FcγRII/εRI, Fc receptor γII/εI; FEV_1_, forced expiratory volume in 1 s; GLUCOLD, Groningen Leiden universities chronic obstructive pulmonary disease; GM-CSF, granulocyte macrophage colony-stimulating factor; GOLD, Global initiative for chronic Obstructive Lung Disease; GPR116, G protein-coupled receptor 116; GWA, genome-wide association; HDL, high-density lipoprotein; HMW, high-molecular weight; ICS, inhaled corticosteroids; IFN-γ, interferon-γ; IgA/E/G/M, immunoglobulin A/E/G/M; IHC, immunohistochemistry; IL-, interleukin-; iNOS, inducible nitric oxide synthase; LAIR-1, leukocyte-associated immunoglobulin-like receptor 1; LDL, low-density lipoprotein; LMW, low-molecular weight; LPS, lipopolysaccharide; ManNAc, *N*-acetylmannosamine; MASP, MBL-associated serine protease; MBL, mannan-binding lectin; MD-2, myeloid differentiation factor 2; Met11, methionine 11; Met11Thr, methionine11threonine; MMP-, matrix metalloproteinase-; NO, nitric oxide; OSCAR, osteoclast-associated receptor; oxLDL, oxidized LDL; PRR, pathogen-recognition receptor; RDS, respiratory distress syndrome; rfhSP-D, 60 kDa recombinant trimeric fragment of SP-D lacking the N-terminal but retaining a part of the collagen region; ROS, reactive oxygen species; RT-PCR, reverse transcription polymerase chain reaction; SDS-PAGE, sodium dodecyl sulfate-polyacrylamide gel electrophoresis; *SFTPD/Sftpd*, SP-D gene; SIRP-α/β, signal-regulatory protein-α/β; SNO-SP-D, S-nitrosothiol SP-D; SNP, single-nucleotide polymorphism; SP-A/B/C/D, surfactant protein A/B/C/D; TGF-β1, transforming growth factor-β1; Th1/2/17, T helper 1/2/17 cell; Thr11, threonine 11; TLR (2/4), toll-like receptor (2/4); TNF-α, tumor necrosis factor-α; UPEC, uropathogenic *Escherichia coli*; UPIa, uroplakin Ia; WB, western blotting.
